# A patient-reported outcome measure comprising the stool frequency and abdominal pain items from the Crohn’s Disease Activity Index: psychometric evaluation in adults with Crohn’s disease

**DOI:** 10.1186/s41687-025-00851-y

**Published:** 2025-02-17

**Authors:** James D. Lewis, Aisha Vadhariya, Sylvia Su, Xian Zhou, Frederick Durand, Ariane K. Kawata, Larissa Stassek, Claudine Clucas, Stefan Schreiber

**Affiliations:** 1 Division of Gastroenterology and Hepatology, University of Pennsylvania, Philadelphia, PA USA; 2 Eli Lilly and Company, 893 S Delaware St., Indianapolis, IN 46225 USA; 3 Syneos Health, Morrisville, NC USA; 4 Evidera, Bethesda, MD USA; 5 Evidera, London, UK; 6 Department of Internal Medicine I, University Hospital Schleswig-Holstein, Christian-Albrechts-University, Kiel, Germany

**Keywords:** Crohn’s disease, Patient reported outcome, Abdominal pain, Stool frequency, Psychometric evaluation

## Abstract

**Background:**

The Stool Frequency (SF) and Abdominal Pain (AP) items from the Crohn’s Disease Activity Index are together referred to as the “Patient Reported Outcome” (PRO). The SF item measures the number of very soft/liquid stools and the AP item measures abdominal pain severity, which are common Crohn’s disease (CD) symptoms that patients consider important to treat. This study evaluated the psychometric properties of both PRO items separately and estimated thresholds for clinical remission in moderately to severely active CD.

**Methods:**

The measurement properties of the PRO items were analyzed using pooled data from VIVID-1 (NCT03926130), a Phase 3, randomized, placebo- and active-controlled study in adults with moderately to severely active CD. Analyses used weekly average scores of the SF and AP items at Weeks 0 (Baseline), 4, 12, and 52. Remission thresholds were estimated using the Patient Global Rating of Severity (PGRS) and Patient Global Impression of Change (PGIC) as primary anchors as well as qualitative evidence from exit interviews.

**Results:**

Data from 1065 participants (mean age: 36.2 years [standard deviation: 13 years]) were analyzed. During the trial, scores improved for both PRO items. Both items demonstrated moderate-to-good test-retest reliability for participants defined as stable based on PGRS and PGIC. Most correlations of related assessments were moderate (0.30≤|ρ*|* <0.70) with SF and moderate-to-large (0.30≤|ρ*|* ≤0.90) with AP. By contrast, as anticipated, both items had weak correlations (|ρ*|* <0.30) with endoscopic and laboratory assessments. The PRO items could discriminate between groups of participants known to differ based on other assessments. The PRO items were able to detect change, as score changes in both items between Baseline and Weeks 12 and 52 differed significantly between most PGRS and PGIC categories. Anchor-based analyses combined with responses from the exit interviews suggested that an SF score of ≤ 3 and an AP score of ≤ 1 could together represent clinical remission.

**Conclusion:**

These results support the reliability, construct-validity, and responsiveness of both PRO items in moderately to severely active CD and confirm previously suggested scores for both items that could represent clinical remission.

**Trial registration:**

Clinicaltrials.gov, NCT03926130. Registered 23 April 2019, https://clinicaltrials.gov/study/NCT03926130.

**Supplementary Information:**

The online version contains supplementary material available at 10.1186/s41687-025-00851-y.

## Abstract (plain language summary)

This study focused on two of the questions (or “items”) from a commonly used questionnaires in Crohn’s disease: the Stool Frequency (SF) item, which counts the number of very soft or liquid stools in a 24-hour period, and the Abdominal Pain (AP) item, which rates severity of abdominal pain in the same time period. We are referring to these two items as the “Patient Reported Outcome” (PRO). This study looked at how well each of these two items did in measuring what they are supposed to measure by using data from people with Crohn’s disease who had participated in a clinical trial. Trial participants answered the SF and AP daily during the trial by using an electronic diary device. Some of the participants were also interviewed to find out when they would consider their disease to be in remission (no longer active). The results showed that both PRO items (SF and AP) can be used by people with Crohn’s disease to accurately measure their very soft or liquid stool frequency and abdominal pain. Further, we estimated which scores on SF and AP could tell us whether someone with Crohn’s disease has achieved remission of their very soft or liquid stools and of their abdominal pain.

**Supplementary Information:**

The online version contains supplementary material available at 10.1186/s41687-025-00851-y.

## Background

Crohn’s disease (CD) is a chronic idiopathic inflammatory bowel disease (IBD) that can affect any segment of the gastrointestinal tract and may result in irreversible damage and disability [1, 2]. Treatment goals have evolved to include both clinical symptom control and the improvement in objective measures of inflammation, such as biomarkers and endoscopic activity [3]. Common symptoms include fatigue, fever, diarrhea, abdominal pain, and weight loss [1, 4–6]. Frequent bowel movements and abdominal pain significantly impact quality of life [7], and they are some of the common CD symptoms that patients consider highly bothersome and important to treat [5, 6, 8, 9].

Recently approved CD therapies included stool frequency and abdominal pain as efficacy endpoints in clinical trials [9, 10]. Resolution of abdominal pain and stool frequency are recommended treatment goals for CD based on Selecting Therapeutic Targets in Inflammatory Bowel Disease (STRIDE-II) recommendations, which also highlight the importance of patient-reported outcomes as standard of measure [11]. Patient reports are particularly critical to measure symptoms that only patients themselves can assess [12]. One measure used to evaluate these symptoms in clinical trials is the Crohn’s Disease Activity Index (CDAI), a composite index comprising three patient-reported items (abdominal pain, stool frequency, and general well-being) alongside physical and laboratory findings [13, 14]. Several measures have been derived from CDAI patient-reported items to specifically assess stool frequency (SF item) and abdominal pain (AP item) [15]. One measure that uses absolute scores for the SF and AP items separately is referred to as the “Patient Reported Outcome” (PRO) [16].

The SF and AP items have largely been evaluated collectively as a combined measure (PRO2) where both items are weighted per CDAI multiplication factors [17]. However, limited evidence exists for the psychometric properties of the SF and AP items when used separately as absolute scores [7, 15, 17–20]. The differences in item scoring and their separate/combined use also warrant additional evidence to confirm the score thresholds representing remission. This study aimed to evaluate the reliability, validity, and responsiveness of both PRO items (SF and AP) separately, and to identify score thresholds representing remission in moderately to severely active CD.

## Methods

### Study population and design

VIVID-1 (NCT03926130) was a Phase 3, multicenter, randomized, double-blind, placebo- and active-controlled study assessing the safety and efficacy of mirikizumab for CD. Participants (18–80 years old) had a confirmed CD diagnosis for ≥ 3 months before Baseline and moderately to severely active disease defined by average daily SF ≥ 4 and/or average daily AP ≥ 2 at Baseline and endoscopic evidence of mucosal inflammation based on a Simple Endoscopic Score for Crohn’s disease (SES-CD) ≥ 7 (or ≥ 4 for those with isolated ileal disease). Participants with intolerance, inadequate response, or loss of response to prior conventional or biologic therapies (or both) were randomly assigned to mirikizumab, ustekinumab, or placebo (6:3:2 ratio) for a total treatment duration of 52 weeks. VIVID-1 methods and primary results have been previously reported [21].

### Assessments

#### PRO items (SF and AP)

Both PRO items were completed daily using an electronic device. Participants were asked to indicate the number of very soft/liquid stools per day per Bristol Stool Form Scale type 6 or 7 (SF) and to rate abdominal pain severity (0=“none,” 1=“mild,” 2=“moderate,” 3=“severe”) (AP) over the past 24 h. Both PRO items were assessed at Baseline and Weeks 4, 12, 16, and 52 by calculating weekly averages using scores from the most recent 7 days in the 12 days before each visit, with at least 4 days of non-missing values.

#### Patient Global Rating of Severity (PGRS)

The PGRS is a single-item instrument assessing the participants’ severity rating of their overall CD symptoms over the past 24 hours (1=“none” to 6=“very severe”). The PGRS was completed daily from the screening visit to Week 52. At Baseline and Weeks 4, 12, 16, and 52, weekly average scores were calculated using the most recent 7 days in the 12 days before each visit, with at least 4 days of non-missing values, and they were rounded to the nearest integer for analysis.

#### Patient Global Impression of Change (PGIC)

The PGIC scale is a single-item instrument assessing the participants’ rating of change in CD symptoms at a given timepoint compared to how they were before they started taking the medicine (Likert scale: 1=“very much better,” 4=“no change,” and 7=“very much worse”). The PGIC was completed at Weeks 4, 8, 12, and 52.

#### Inflammatory Bowel Disease Questionnaire (IBDQ)

The IBDQ is a 32-item instrument [22] that measures four domains of participants’ lives over the past 2 weeks: symptoms directly related to the primary bowel disturbance (e.g., loose stools and abdominal pain; 10 items), systemic symptoms (e.g., weight loss and altered sleep patterns; 5 items), emotional function (12 items), and social function (5 items). Scores (Likert scale: 1=“a very severe problem” to 7=“not a problem at all”) are summed to give a total score (range, 32–224; a higher score indicates a better quality of life). IBDQ item 1 asks participants to rate their bowel movement frequency over the past 2 weeks from 1 (”more frequent than ever”) to 7 (”normal, no increase”). The IBDQ was completed at Baseline, Week 12, and Week 52. IBDQ response was defined as a ≥ 16-point improvement (increase) from Baseline in IBDQ total score and IBDQ remission as an IBDQ total score of ≥ 170 [23, 24].

#### 36-Item Short Form Health Survey (SF-36)

The SF-36 includes 36 items measuring eight health domains: physical functioning, social functioning, role limitations due to physical problems, role limitations due to emotional problems, mental health, energy/fatigue, bodily pain, and general health perception. Each item has 3–6 response options and scores (range, 0–100) can be calculated for each domain, with higher scores indicating better health status. The version used in the study (SF-36 v2 ‘acute’) has a 1-week recall period. The SF-36 was completed at Baseline, Week 12, and Week 52.

#### EQ-5D-5 L

The EQ-5D-5L assesses health status across five dimensions (mobility, self-care, usual activities, pain/discomfort, and anxiety/depression), each with five levels of response (1=”no problems” to 5=”extreme problems”). The recall period is “today.” The pain/discomfort domain was of particular interest for the psychometric evaluation of the AP item as it was expected to relate to abdominal pain in CD. The EQ-5D-5 L was completed at Baseline, Week 12, and Week 52.

#### SES-CD

The SES-CD is a CD-specific endoscopic scoring system based on four endoscopic variables assessed in five bowel segments (scale: 0–3 per variable and bowel segment). The sum of the scores for each endoscopic variable (0–11 for presence and severity of intestinal luminal narrowing and 0–15 for the rest) leads to an overall range of 0–56, with higher scores indicating more severe disease. The SES-CD was completed at screening, Week 12, and Week 52. Centrally-read SES-CD scores from the screening endoscopy were considered the baseline for endoscopic response/remission endpoints.

#### Inflammation markers

The concentrations of high-sensitivity C-reactive protein (hsCRP) from blood samples and fecal calprotectin from stool samples assessed inflammation levels at Baseline and at Weeks 4, 8 (only hsCRP), 12, 16, 28, 44, and 52.

### Psychometric evaluation

The present psychometric analyses used individual participant data from VIVID-1 pooled across treatment groups. All variables were summarized descriptively.

#### Test-retest reliability

Test-retest reliability of the PRO items was evaluated by calculating intraclass correlation coefficients (ICCs) and difference in scores (using paired t-tests) between Baseline and Week 4 in subgroups of stable participants. Two subgroups of stable participants were defined as follows: (1) those with an unchanged PGRS score between Baseline and Week 4, and (2) those with a response of “no change” on the PGIC at Week 4. Good test-retest reliability was supported by ICCs ≥ 0.70; ICCs > 0.90 indicated excellent reliability [25].

#### Convergent and discriminant validity

Convergent and discriminant validity of the PRO items (SF/AP) were assessed at Baseline, Week 12, and Week 52. Convergent validity was assessed by calculating Spearman correlations between the SF item and PGRS, IBDQ Bowel Symptom domain, IBDQ item 1 (bowel movement frequency), and IBDQ total score; as well as between the AP item and PGRS, IBDQ item 13 (abdominal pain frequency), EQ-5D-5 L Pain/Discomfort, and SF-36 Bodily Pain. Discriminant validity was assessed by calculating Spearman correlations between SF and AP and SES-CD, hsCRP, and fecal calprotectin. Based on its absolute value, a correlation < 0.30 was considered as weak, ≥ 0.30 to < 0.70 as moderate, ≥ 0.70 to ≤ 0.90 as large, and > 0.90 as very large [26]. Moderate or stronger correlations (|ρ| ≥0.30) were expected for the convergent validity assessments and weak correlations (|ρ| <0.30) were expected for the discriminant validity assessments.

#### Known-groups validity

Known-groups validity of the PRO items was evaluated by comparing PRO item (SF/AP) scores between subgroups based on PGRS categories (levels of response), median split IBDQ Bowel Symptom domain score (SF only), and EQ-5D-5 L Pain/Discomfort categories (AP only) at Baseline, Week 12, and Week 52. Mean scores on the PRO items (SF/AP) were compared between individual subgroups using analysis of variance (ANOVA) with Scheffé’s correction for post-hoc pairwise comparisons [27]. The ANOVA models included each PRO item (SF/AP) score as the dependent variable and the known-group variable as the independent variable. Participants with more severe CD symptoms based on these other measures were expected to report greater stool frequency and abdominal pain symptom severity (higher SF/AP scores). Pairwise effect sizes (Cohen’s d) were derived as the mean difference between two groups divided by the pooled standard deviation (SD), to measure the standardized differences between groups. It is suggested that d = 0.2 can be considered a small effect size, 0.5 a medium effect size, and 0.8 a large effect size [28].

#### Ability to detect change (responsiveness)

Spearman correlations for change scores from Baseline to Week 12 and Week 52 were calculated between the changes in PRO items (SF/AP) and corresponding changes in potential anchor measures (PGRS, PGIC, IBDQ Bowel Symptom domain [SF only], IBDQ item 1 [bowel movement frequency; SF only], IBDQ item 13 [abdominal pain frequency; AP only], and IBDQ total score). The responsiveness of the PRO items (SF/ AP) was also evaluated by comparing mean changes in the SF/AP scores from Baseline to Weeks 12 and 52 with changes between pre-defined anchor groups in the same period using one-way analysis of covariance (ANCOVA) with Scheffé’s correction for post-hoc pairwise comparisons [27] and controlling for the SF or AP score at Baseline. Pairwise effect sizes (Cohen’s d) were derived as described in the previous paragraph.

The anchor groups were pre-defined by PGRS average change, PGIC categories, IBDQ response, IBDQ remission, ≥ 8-point increase from Baseline in IBDQ Bowel Symptom domain score [29] (SF only), and ≥ 1-point increase in IBDQ item 1 (bowel movement frequency) score (SF only), ≥ 1-point increase in IBDQ item 13 (abdominal pain frequency) score (AP only).

#### Interpretation of remission by PRO

Using an anchor-based approach, the remission definitions for PRO (SF/AP) scores were estimated based on response criteria for the anchor measures. Analyses examined SF/AP scores at Week 12 and Week 52 to validate the pre-specified endpoint definition for clinical remission by PRO (unweighted weekly averages of ≤ 3 for SF and ≤ 1 for AP, with neither score being worse than Baseline). PGRS and PGIC were used as primary anchors for both SF and AP. The supplemental anchors for the SF item were IBDQ remission, IBDQ Bowel Symptom domain, and IBDQ item 1 (bowel movement frequency). The supplemental anchors for the AP item were IBDQ remission, IBDQ item 13 (abdominal pain frequency), and EQ-5D-5 L Pain/Discomfort. Mean, SD, and percentile groups (10th, 25th [quartile 1], 50th [quartile 2, median], 75th [quartile 3], and 90th) for SF and AP scores were reported for participants in each of the anchor groups. The estimates were used to identify thresholds that could indicate remission.

Anchor-based methods were supplemented with cumulative distribution function (CDF) and probability density function (PDF) plots, estimated using kernel density estimation curves, to examine the distribution of SF and AP scores by PGRS score and PGIC categories. The cumulative proportion (CDF) or probability density (PDF) were shown across a range of possible responder definitions as defined by PGRS and PGIC.

To provide qualitative evidence for the proposed clinical remission thresholds for the PRO items, exit interviews were held with a subgroup of participants from VIVID-1 [30]. Interview participants were required to have completed all study treatment period procedures. Interviews included open-ended exploratory questions and cognitive debriefing. A subset of exit interview participants was queried about their thoughts on proposed remission thresholds for the PRO items. Participants were asked if they would consider having ≤ 3 type 6 or 7 bowel movements and mild to no abdominal pain in 24 h on most days as being “in remission.” Ethical approval for the clinical trial protocol addendum and additional written informed consent (related to the exit interviews) were obtained before interview completion.

## Results

### Baseline demographic and clinical characteristics

The analysis included data from 1065 participants. The mean participant age was 36.2 years (SD: 13.0) (Table 1). Just over half of the participants (55%) were male and 71% were White. Over two thirds (70%) were 17–40 years old at time of CD diagnosis and 86% had had the disease for at least 1 year. Mean duration of CD was 7.4 years. Around a third (31%) of the participants were using corticosteroids at Baseline and almost half (49%) had prior failure to biologic therapy.

**Table 1:** Baseline demographic and clinical characteristics in VIVID-1

Characteristic	*N* = 1065
Age (years), mean (SD)	36.2 (13.0)
Sex, n (%)	
Male	587 (55)
Female	478 (45)
Race, n (%)	
White	753 (71)
Black or African American	23 (2)
Asian	264 (25)
American Indian/Alaska Native	6 (1)
Multiple	4 (0)
Missing	15 (1)
Age at CD diagnosis, n (%)	
< 10 years	6 (1)
≥ 10 to < 17 years	107 (10)
≥ 17 to < 40 years	747 (70)
≥ 40 years	204 (19)
Missing	1 (0)
Duration of CD, n (%)	
< 1 year	147 (14)
≥ 1 to < 5 years	392 (37)
≥ 5 years	525 (49)
Missing	1 (0)
Corticosteroid use, n (%)	325 (31)
Prior biologic therapy failure, n (%)	517 (49)

Abbreviations: CD, Crohn’s disease; SD, standard deviation

### Distribution of PRO (SF/AP) scores

**Fig. 1:**
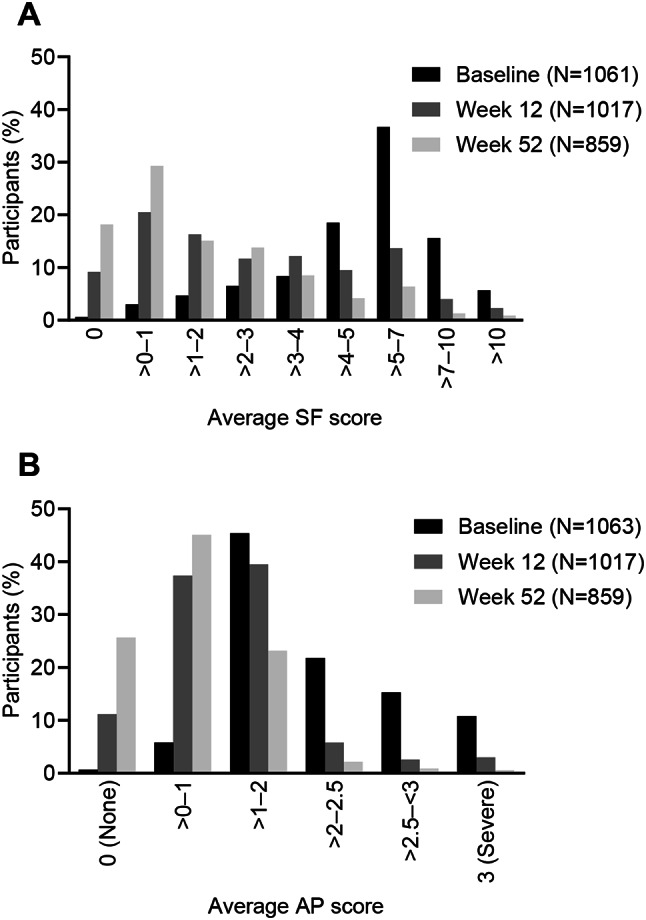
PRO score distributions per item (SF and AP). (**A**) Average weekly scores for SF. (**B**) Average weekly scores for AP. Abbreviations: AP, Abdominal Pain; PRO, Patient Reported Outcome; SF, Stool Frequency

SF scores at Baseline ranged from 0.0 to 30.6, with a mean score of 5.7 (SD: 3.0); over half of participants (58%) had average scores > 5 at Baseline (Fig. 1). By Week 12, the mean score decreased to 3.1 (SD: 2.9). By Week 52, most participants (63%) had scores ≤ 2, and the mean score decreased further to 1.9 (SD: 2.3). AP scores at Baseline ranged from 0 (none) to 3 (severe), with a mean score of 2.1 (SD: 0.6); nearly half of participants (48%) had average scores > 2 at Baseline. At Week 12, scores were more evenly distributed, and the mean score decreased to 1.2 (SD: 0.8). By Week 52, most participants (71%) had scores ≤ 1, with a mean score of 0.8 (SD: 0.7). No extreme ceiling and floor effects were observed for either item at any of the analyzed timepoints.

### Test-retest reliability

For SF, 417 participants were defined as stable at Week 4 based on the PGRS (ICC = 0.85) and 266 for PGIC (ICC = 0.79). For AP, 418 participants were defined as stable based on PGRS (ICC = 0.82) and 266 for PGIC (ICC = 0.69) (Table 2). ICCs indicated good reliability.

**Table 2:** Test-retest reliability of the PRO (SF and AP) items

	*N*	Baseline mean (SD)	Week 4 mean (SD)	Mean difference	ICC
Stable on PGRS^a^					
SF score	417	5.7 (2.93)	4.7 (3.00)	−0.9	0.85
AP score	418	2.0 (0.61)	1.9 (0.64)	−0.1	0.82
Stable on PGIC^b^					
SF score	266	5.9 (3.17)	4.9 (3.31)	−0.9	0.79
AP score	266	2.1 (0.62)	1.8 (0.69)	−0.3	0.69

^a^Participants whose PGRS average score (rounded to the nearest integer) did not change between Baseline and Week 4

^b^Participants who reported “no change” on the PGIC at Week 4 (PGIC = 4)

Abbreviations: AP, Abdominal Pain; ICC, intraclass correlation coefficient; PGIC, Patient Global Impression of Change; PGRS, Patient Global Rating of Severity; PRO, Patient Reported Outcome; SD, standard deviation; SF, Stool Frequency

### Convergent and discriminant validity

At Baseline, SF was moderately correlated with IBDQ item 1 for bowel movement frequency (*|*ρ*|*=0.35) and weakly correlated (|ρ*|*=0.03–0.25) with all other measures (Table 3). At Weeks 12 and 52, SF showed moderate correlations with all measures as hypothesized (|ρ*|*=0.46–0.54 at Week 12 and 0.44–0.54 at Week 52). Correlations of SF with endoscopic/laboratory assessments (SES-CD, hsCRP, and fecal calprotectin) were weak across timepoints (|ρ*|*=0.04–0.20 at Baseline, 0.14–0.19 at Week 12, and 0.13–0.23 at Week 52).

**Table 3:** Convergent and discriminant validity of the PRO (SF and AP) items

	SF score		AP score	
	N	ρ (95% CI)^a^	N	ρ (95%CI)^a^
Baseline				
PGRS average	1060	0.15 (0.09, 0.21)	1062	0.83 (0.80, 0.84)
IBDQ Bowel Symptom domain	1057	−0.25 (− 0.30, − 0.19)	1059	–
IBDQ item 1 (bowel movement frequency)	1057	−0.35 (− 0.40, − 0.29)	1059	–
IBDQ item 13 (abdominal pain frequency)	1057	–	1059	−0.41 (− 0.46, − 0.36)
IBDQ total score	1057	−0.21 (− 0.26, − 0.15)	1059	−0.31 (− 0.37, − 0.26)
SF-36 Bodily Pain domain	1057	–	1059	−0.46 (− 0.50, − 0.41)
EQ-5D-5 L Pain/Discomfort	1057	–	1059	0.45 (0.40, 0.50)
SES-CD	1061	0.20 (0.14, 0.26)	1063	−0.03 (− 0.09, 0.03)
hsCRP (mg/L)	1061	0.07 (0.01, 0.13)	1063	0.05 (− 0.01, 0.11)
Fecal calprotectin (mg/kg)	832	0.04 (− 0.03, 0.11)	834	0.11 (0.04, 0.18)
Week 12				
PGRS average	1016	0.54 (0.49, 0.58)	1016	0.86 (0.84, 0.87)
IBDQ Bowel Symptom domain	1009	−0.54 (− 0.58, − 0.49)	1009	–
IBDQ item 1 (bowel movement frequency)	1009	−0.47 (− 0.52, − 0.42)	1009	–
IBDQ item 13 (abdominal pain frequency)	1009	–	1009	−0.63 (− 0.67, − 0.59)
IBDQ total score	1009	−0.46 (− 0.51, − 0.41)	1009	−0.57 (− 0.61, − 0.53)
SF-36 Bodily Pain domain	1009	–	1009	−0.63 (− 0.66, − 0.59)
EQ-5D-5 L Pain/Discomfort	1009	–	1009	0.55 (0.50, 0.59)
SES-CD	925	0.19 (0.12, 0.25)	925	0.07 (0.00, 0.13)
hsCRP (mg/L)	987	0.14 (0.07, 0.20)	987	0.14 (0.08, 0.20)
Fecal calprotectin (mg/kg)	278	0.14 (0.02, 0.25)	278	0.18 (0.07, 0.29)
Week 52				
PGRS average	859	0.52 (0.46, 0.56)	859	0.80 (0.78, 0.83)
IBDQ Bowel Symptom domain	836	−0.54 (− 0.59, − 0.49)	836	–
IBDQ item 1 (bowel movement frequency)	835	−0.44 (− 0.50, − 0.39)	835	–
IBDQ item 13 (abdominal pain frequency)	835	–	835	−0.60 (− 0.64, − 0.55)
IBDQ total score	835	−0.47 (− 0.52, − 0.42)	835	−0.49 (− 0.54, − 0.44)
SF-36 Bodily Pain domain	835	–	835	−0.57 (− 0.61, − 0.52)
EQ-5D-5 L Pain/Discomfort	835	–	835	0.50 (0.45, 0.55)
SES-CD	843	0.22 (0.15, 0.28)	843	0.09 (0.02, 0.15)
hsCRP (mg/L)	828	0.23 (0.16, 0.29)	828	0.17 (0.11, 0.24)
Fecal calprotectin (mg/kg)	281	0.13 (0.01, 0.24)	281	0.08 (− 0.04, 0.19)

^a^Correlations were hypothesized to be moderate (|ρ|=0.3–0.7) for PGRS (SF and AP), IBDQ Bowel Symptom domain (SF), IBDQ item 1 (bowel movement frequency; SF), IBDQ item 13 (abdominal pain frequency; AP), SF-36 Bodily Pain domain (AP), EQ-5D-5 L Pain/Discomfort (AP); low to moderate (|ρ|=0.3–0.5) for IBDQ total score (SF and AP); and weak (|ρ| <0.3) for SES-CD, hsCRP, and fecal calprotectin (SF and AP)

Abbreviations: AP, Abdominal Pain; CI, confidence interval; hsCRP, high-sensitivity C-reactive protein; IBDQ, Inflammatory Bowel Disease Questionnaire; PGRS, Patient Global Rating of Severity; PRO, Patient Reported Outcome; SES-CD, Simple Endoscopic Score for Crohn’s Disease; SF, Stool Frequency; SF-36, Medical Outcomes Study 36-Item Short Form Health Survey

At Baseline, AP showed a strong correlation with PGRS (*|*ρ*|*=0.83) and moderate correlations with all other measures as hypothesized (|ρ*|*=0.31–0.46) (Table 3). Similar findings were found at Weeks 12 and 52. Correlations of AP with endoscopic/laboratory assessments (SES-CD, hsCRP, and fecal calprotectin) were weak at all timepoints (|ρ*|*=0.03–0.11 at Baseline, 0.07–0.18 at Week 12, and 0.08–0.17 at Week 52).

### Known-groups validity

Overall, participants with higher (more severe) PGRS scores tended to have higher mean SF and AP scores at Baseline, Week 12, and Week 52 (Table 4, Suppl. Tables 1–2). For SF, while absolute effect sizes for between-group comparisons ranged from small to medium (Cohen’s d: 0.1–0.7) at Baseline, they were mostly medium to large at Weeks 12 and 52; for AP, they were mostly large at all timepoints. Because our population consisted of individuals with moderately to severely active CD, most had moderate to severe PGRS scores at Baseline (Table 4). Baseline results involving the “none” PGRS category should be interpreted with caution due to the small group size (*N* = 3). As symptoms generally improved during the study, the pattern of PGRS scores shifted towards response categories that indicated less severe disease activity at Weeks 12 and 52 (Suppl. Tables 1–2). Participants with increased severity of EQ-5D-5 L Pain/Discomfort had higher AP scores at all timepoints (Suppl. Table 3), with mostly medium to large absolute size effects.

**Table 4:** Known-groups validity of the PRO (SF and AP) items by PGRS at Baseline

PGRS scores	1 = None	2 = Very mild	3 = Mild	4 = Moderate	5 = Severe	6 = Very severe
SF scores	*N* = 3	*N* = 14	*N* = 84	*N* = 455	*N* = 415	*N* = 89
Mean (SD)	5.7 (1.1)	5.1 (1.6)	5.6 (1.6)	5.2 (2.5)	5.9 (3.0)	7.3 (5.1)
Median (range)	6.0 (4.4, 6.6)	5.3 (1.4, 7.6)	5.8 (1.0, 10.3)	5.1 (0.0, 23.4)	5.9 (0.0, 23.9)	6.3 (0.0, 30.6)
Effect size						
vs. 1 = None	–	−0.4	−0.0	−0.2	0.1	0.3
vs. 2 = Very mild	–	–	0.3	0.0	0.3	0.5
vs. 3 = Mild	–	–	–	−0.2	0.1	0.5
vs. 4 = Moderate	–	–	–	–	0.2	0.7
vs. 5 = Severe	–	–	–	–	–	0.4
LSM (SE)	5.7 (1.7)	5.1 (0.8)	5.6 (0.3)	5.2 (0.1)	5.9 (0.1)	7.3 (0.3)
LSMD (CI)						
vs. 1 = None	–	−0.5 (− 6.8, 5.7)	−0.1 (− 5.8, 5.7)	−0.4 (− 6.1, 5.3)	0.2 (− 5.5, 5.9)	1.7 (− 4.1, 7.4)
vs. 2 = Very mild	–	–	0.5 (− 2.4, 3.3)	0.1 (− 2.6, 2.8)	0.8 (− 1.9, 3.4)	2.2 (− 0.6, 5.0)
vs. 3 = Mild	–	–	–	−0.4 (− 1.5, 0.8)	0.3 (− 0.9, 1.5)	1.7 (0.2, 3.2)*
vs. 4 = Moderate	–	–	–	–	0.7 (0.0, 1.3)*	2.1 (1.0, 3.2)****
vs. 5 = Severe	–	–	–	–	–	1.4 (0.3, 2.6)**
AP scores	*N* = 3	*N* = 14	*N* = 85	*N* = 457	*N* = 415	*N* = 88
Mean (SD)	0.1 (0.2)	0.7 (0.4)	1.1 (0.4)	1.9 (0.3)	2.4 (0.4)	2.9 (0.3)
Median (range)	0.0 (0.0, 0.3)	0.7 (0.0, 1.1)	1.1 (0.0, 2.1)	2.0 (0.0, 2.9)	2.4 (0.6, 3.0)	3.0 (1.1, 3.0)
Effect size						
vs. 1 = None	–	1.8	2.7	5.3	5.9	10.2
vs. 2 = Very mild	–	–	1.1	3.5	4.4	7.5
vs. 3 = Mild	–	–	–	2.2	3.3	5.2
vs. 4 = Moderate	–	–	–	–	1.4	2.8
vs. 5 = Severe	–	–	–	–	–	1.1
LSM (SE)	0.1 (0.2)	0.7 (0.1)	1.1 (0.0)	1.9 (0.0)	2.4 (0.0)	2.9 (0.0)
LSMD (CI)						
vs. 1 = None	–	0.6 (− 0.2, 1.4)	1.0 (0.3, 1.7)***	1.8 (1.1, 2.5)****	2.3 (1.6, 3.0)****	2.8 (2.0, 3.5)****
vs. 2 = Very mild	–	–	0.4 (0.1, 0.8)**	1.2 (0.9, 1.5)****	1.7 (1.4, 2.1)****	2.2 (1.8, 2.5)****
vs. 3 = Mild	–	–	–	0.8 (0.6, 0.9)****	1.3 (1.2, 1.5)****	1.7 (1.6, 1.9)****
vs. 4 = Moderate	–	–	–	–	0.5 (0.4, 0.6)****	0.9 (0.8, 1.1)****
vs. 5 = Severe	–	–	–	–	–	0.4 (0.3, 0.6)****

*p < 0.05, ** *p* < 0.01, *** *p* < 0.001, **** *p* < 0.0001. Effect size was estimated using Cohen’s d (mean difference divided by the pooled SD). Pairwise LSMD (CI) and *p*-values were adjusted using Scheffe’s correction

Abbreviations: AP, Abdominal Pain; CI, confidence interval; LSM, least square mean; LSMD, least square mean difference; PGRS, Patient Global Rating of Severity; PRO, Patient Reported Outcome; SD, standard deviation; SE, standard error; SF, Stool Frequency

### Responsiveness

Moderate to large correlations were observed between the changes, from Baseline to Weeks 12 and 52, in SF and AP and the changes in PGRS (|ρ*|*=0.52–0.40 for SF and|ρ*|*=0.85–0.82 for AP), IBDQ total score (|ρ*|*=0.42–0.31 for SF and|ρ*|*=0.49–0.40 for AP), IBDQ Bowel Symptom domain (SF only;|ρ*|*=0.46–0.35), IBDQ item 1 (bowel movement frequency; SF only;|ρ*|*=0.43–0.38), and IBDQ item 13 (abdominal pain frequency; AP only;|ρ*|*=0.51–0.47), as well as PGIC score (Suppl. Table 4).

Improvement in PGRS corresponded to greater improvement (larger negative change scores) on SF and AP from Baseline to Weeks 12 and 52. Differences in mean SF and AP change were statistically significant between most PGRS categories for SF and between all PGRS categories for AP (Table 5). For SF, absolute effect sizes for between-group comparisons were small to large for change from Baseline to Weeks 12 and 52; for AP, they were mostly large (Cohen’s d > 1.0). Improvements in scores of SF and AP were associated with lower (improved) PGIC, IBDQ response (≥ 16-point increase), IBDQ remission (score ≥ 170), IBDQ Bowel Symptom domain score response (≥ 8-point increase; SF only), improvement on IBDQ item 1 (≥ 1-point increase in bowel movement frequency; SF only), and IBDQ item 13 (≥ 1-point increase in abdominal pain frequency; AP only) from Baseline to Weeks 12 and 52 (Suppl. Tables 5–7).

**Table 5:** Responsiveness of the PRO (SF and AP) items by PGRS change from baseline to weeks 12 and 52

PGRS change from Baseline	≥ 4-point improvement	3-point improvement	2-point improvement	1-point improvement	No change	≥ 1-point worsening
SF score change from Baseline to Week 12	*N* = 47	*N* = 126	*N* = 220	*N* = 315	*N* = 267	*N* = 36
Mean (SD)	−6.0 (4.0)	−4.1 (2.3)	−3.7 (2.5)	−2.6 (2.4)	−1.2 (1.7)	0.5 (3.9)
Median (range)	−5.7 (− 17.9, 0.6)	−4.0 (− 11.4, 0.6)	−3.6 (− 12.0, 3.7)	−2.3 (− 16.3, 4.3)	−0.9 (− 7.4, 5.1)	−0.1 (− 5.4, 16.0)
Effect size						
vs. ≥4-point improvement	–	−0.7	−0.8	−1.3	−2.2	−1.6
vs. 3-point improvement	–	–	−0.2	−0.6	−1.5	−1.7
vs. 2-point improvement	–	–	–	−0.5	−1.2	−1.5
vs. 1-point improvement	–	–	–	–	−0.7	−1.2
vs. no change	–	–	–	–	–	−0.8
LSM (SE)	−5.5 (0.3)	−4.3 (0.2)	−3.7 (0.1)	−2.5 (0.1)	−1.2 (0.1)	0.5 (0.3)
LSMD (CI)						
vs. ≥4-point improvement	–	−1.2 (− 2.3, − 0.1)	−1.8 (− 2.8, − 0.7)	−3.0 (− 4.0, − 1.9)	−4.3 (− 5.4, − 3.3)	−6.0 (− 7.5, − 4.5)
vs. 3-point improvement	–	–	−0.5 (− 1.3, 0.2)	−1.8 (− 2.5, − 1.1)	−3.1 (− 3.8, − 2.4)	−4.8 (− 6.0, − 3.5)
vs. 2-point improvement	–	–	–	−1.2 (− 1.8, − 0.6)	−2.6 (− 3.2, − 2.0)	−4.2 (− 5.4, − 3.0)
vs. 1-point improvement	–	–	–	–	−1.4 (− 1.9, − 0.8)	−3.0 (− 4.2, − 1.8)
vs. no change	–	–	–	–	–	−1.7 (− 2.8, − 0.5)
SF score change from Baseline to Week 52	*N* = 97	*N* = 222	*N* = 213	*N* = 204	*N* = 106	*N* = 13
Mean (SD)	−5.6 (3.8)	−4.7 (2.8)	−3.8 (2.5)	−2.9 (2.3)	−2.0 (2.3)	−1.0 (2.7)
Median (range)	−5.3 (− 20.9, − 0.1)	−4.6 (− 19.6, 0.3)	−3.7 (− 12.3, 1.3)	−2.6 (− 12.0, 7.3)	−1.9 (− 8.9, 3.7)	−1.0 (− 7.1, 2.4)
Effect size						
vs. ≥4-point improvement	–	−0.3	−0.6	−0.9	−1.2	−1.2
vs. 3-point improvement	–	–	−0.3	−0.7	−1.0	−1.3
vs. 2-point improvement	–	–	–	−0.4	−0.8	−1.1
vs. 1-point improvement	–	–	–	–	−0.4	−0.8
vs. no change	–	–	–	–	–	−0.4
LSM (SE)	−5.2 (0.2)	−4.8 (0.1)	−3.8 (0.1)	−3.0 (0.1)	−2.0 (0.2)	−0.7 (0.5)
LSMD (CI)						
vs. ≥4-point improvement	–	−0.4 (− 1.2, 0.3)	−1.4 (− 2.2, − 0.7)	−2.2 (− 2.9, − 1.5)	−3.2 (− 4.0, − 2.3)	−4.5 (− 6.3, − 2.8)
vs. 3-point improvement	–	–	−1.0 (− 1.6, − 0.4)	−1.8 (− 2.3, − 1.2)	−2.7 (− 3.4, − 2.0)	−4.1 (− 5.8, − 2.4)
vs. 2-point improvement	–	–	–	−0.8 (− 1.4, − 0.2)	−1.7 (− 2.5, − 1.0)	−3.1 (− 4.8, − 1.4)
vs. 1-point improvement	–	–	–	–	−1.0 (− 1.7, − 0.3)	−2.3 (− 4.0, − 0.6)
vs. no change	–	–	–	–	–	−1.4 (− 3.1, 0.4)
AP score change from Baseline to Week 12	*N* = 47	*N* = 126	*N* = 222	*N* = 315	*N* = 267	*N* = 36
Mean (SD)	−2.4 (0.4)	−1.8 (0.5)	−1.3 (0.4)	−0.7 (0.4)	−0.2 (0.4)	0.4 (0.6)
Median (range)	−2.6 (− 3.0, − 1.4)	−1.9 (− 3.0, − 0.1)	−1.3 (− 2.6, − 0.1)	−0.7 (− 2.4, 0.7)	0.0 (− 2.1, 0.6)	0.4 (− 2.0, 1.4)
Effect size						
vs. ≥4-point improvement	–	−1.3	−2.5	−3.7	−5.8	−5.5
vs. 3-point improvement	–	–	−1.0	−2.3	−3.8	−4.2
vs. 2-point improvement	–	–	–	−1.2	−2.7	−3.5
vs. 1-point improvement	–	–	–	–	−1.3	−2.3
vs. no change	–	–	–	–	–	−1.3
LSM (SE)	−2.3 (0.1)	−1.7 (0.0)	−1.3 (0.0)	−0.7 (0.0)	−0.2 (0.0)	0.3 (0.1)
LSMD (CI)						
vs. ≥4-point improvement	–	−0.5 (− 0.8, − 0.3)	−1.0 (− 1.2, − 0.8)	−1.5 (− 1.8, − 1.3)	−2.1 (− 2.3, − 1.8)	−2.6 (− 2.9, − 2.3)
vs. 3-point improvement	–	–	−0.5 (− 0.6, − 0.3)	−1.0 (− 1.1, − 0.8)	−1.5 (− 1.7, − 1.4)	−2.1 (− 2.3, − 1.8)
vs. 2-point improvement	–	–	–	−0.5 (− 0.7, − 0.4)	−1.1 (− 1.2, − 0.9)	−1.6 (− 1.9, − 1.3)
vs. 1-point improvement	–	–	–	–	−0.5 (− 0.6, − 0.4)	−1.1 (− 1.3, − 0.8)
vs. no change	–	–	–	–	–	−0.5 (− 0.8, − 0.3)
AP score change from Baseline to Week 52	*N* = 97	*N* = 222	*N* = 215	*N* = 205	*N* = 106	*N* = 13
Mean (SD)	−2.4 (0.4)	−1.8 (0.5)	−1.3 (0.5)	−0.8 (0.5)	−0.4 (0.6)	0.3 (0.4)
Median (range)	−2.4 (− 3.0, − 0.6)	−2.0 (− 3.0, 0.0)	−1.3 (− 3.0, − 0.1)	−0.8 (− 3.0, 0.4)	−0.1 (− 2.6, 0.9)	0.3 (− 0.3, 1.4)
Effect size						
vs. ≥4-point improvement	–	−1.3	−2.4	−3.5	−3.7	−6.2
vs. 3-point improvement	–	–	−1.1	−2.2	−2.8	−4.7
vs. 2-point improvement	–	–	–	−1.1	−1.8	−3.5
vs. 1-point improvement	–	–	–	–	−0.7	−2.3
vs. no change	–	–	–	–	–	−1.2
LSM (SE)	−2.3 (0.0)	−1.8 (0.0)	−1.3 (0.0)	−0.9 (0.0)	−0.5 (0.0)	0.2 (0.1)
LSMD (CI)						
vs. ≥4-point improvement	–	−0.5 (− 0.6, − 0.3)	−1.0 (− 1.1, − 0.8)	−1.4 (− 1.6, − 1.2)	−1.8 (− 2.0, − 1.6)	−2.4 (− 2.9, − 2.0)
vs. 3-point improvement	–	–	−0.5 (− 0.6, − 0.4)	−0.9 (− 1.1, − 0.8)	−1.3 (− 1.5, − 1.1)	−2.0 − 2.4, − 1.5)
vs. 2-point improvement	–	–	–	−0.4 (− 0.6, − 0.3)	−0.8 (− 1.0, − 0.6)	−1.5 (− 1.9, − 1.0)
vs. 1-point improvement	–	–	–	–	−0.4 (− 0.6, − 0.2)	−1.0 (− 1.5, − 0.6)
vs. no change	–	–	–	–	–	−0.6 (− 1.1, − 0.2)

Effect size was estimated using Cohen’s d (mean difference divided by the pooled SD). Pairwise LSMD (CI) were adjusted using Scheffe’s correction

Abbreviations: AP, Abdominal Pain; CI, confidence interval; LSM, least square mean; LSMD, least square mean difference; PGRS, Patient Global Rating of Severity; PRO, Patient Reported Outcome; SD, standard deviation; SE, standard error; SF, Stool Frequency

### Interpretation of remission by PRO

At Week 12, the median SF score estimate was 1.3 for the PGRS “very mild” category and 1.6 for the PGIC “much better” category (Table 6). At Week 52, it was 0.9 for the PGRS “very mild” category and 1.3 for the PGIC “much better” category (Suppl. Table 8). The median SF estimate was also within the range of 1–2 among participants with meaningful improvement according to the supplemental anchors (Suppl. Tables 9–10). Additionally, at Week 12, the median SF estimates were 2.0 for the PGRS “mild” category, 3.7 for the PGRS “moderate” category, and 3.1 for the PGIC “a little better” category (Table 6). The supplemental anchors also had similar findings (Suppl. Table 9). This suggests that a threshold of 3 in SF score could represent mild to moderate symptom severity.

**Table 6:** Anchor-based estimates for SF and AP by PGRS and PGIC at Week 12

Anchors	SF item							AP item						
	*N*	Mean (SD)	Percentile					*N*	Mean (SD)	Percentile				
			10th	25th (Q1)	50th (Q2)	75th (Q3)	90th			10th	25th (Q1)	50th (Q2)	75th (Q3)	90th
PGRS														
1 = None	107	1.0 (1.6)	0.0	0.0	0.3	1.3	2.6	107	0.1 (0.2)	0.0	0.0	0.0	0.1	0.3
2 = Very mild	211	1.8 (1.8)	0.0	0.4	1.3	2.7	4.3	211	0.7 (0.4)	0.0	0.3	0.8	1.0	1.0
3 = Mild	274	2.4 (2.0)	0.1	1.0	2.0	3.7	5.3	274	1.1 (0.3)	0.7	1.0	1.0	1.3	1.4
4 = Moderate	289	3.9 (2.7)	0.7	2.0	3.7	5.4	7.0	289	1.7 (0.5)	1.0	1.6	2.0	2.0	2.0
5 = Severe	109	5.8 (4.3)	1.1	3.7	5.0	6.8	10.1	109	2.2 (0.6)	2.0	2.0	2.1	2.6	3.0
6 = Very Severe	26	6.6 (3.8)	2.3	4.0	6.7	7.4	11.1	26	2.8 (0.4)	2.4	2.9	3.0	3.0	3.0
PGIC														
1 = Very much better	154	1.4 (1.8)	0.0	0.0	0.7	2.1	4.0	154	0.6 (0.6)	0.0	0.0	0.5	1.0	1.1
2 = Much better	325	2.1 (1.9)	0.1	0.7	1.6	3.1	4.6	325	1.0 (0.7)	0.0	0.4	1.0	1.4	2.0
3 = A little better	308	3.5 (3.0)	0.6	1.4	3.1	4.8	6.4	308	1.4 (0.7)	0.4	1.0	1.4	2.0	2.1
4 = No change	160	4.7 (3.4)	0.9	2.4	4.6	6.3	7.7	160	1.7 (0.8)	0.4	1.1	2.0	2.1	2.7
5 = A little worse	33	5.2 (3.8)	2.0	3.1	4.1	6.0	11.1	33	1.9 (0.6)	1.0	1.7	2.0	2.1	2.6
6 = Much worse	21	5.3 (2.6)	1.4	3.9	5.4	7.0	7.9	21	2.0 (0.7)	1.0	1.6	2.0	2.3	3.0
7 = Very much worse	8	8.7 (5.2)	3.7	6.0	7.4	9.3	20.7	8	2.1 (0.7)	1.0	1.7	2.1	2.6	2.9

Abbreviations: AP, Abdominal Pain; PGRS, Patient Global Rating of Severity; PGIC, Patient Global Impression of Change; Q, quartile; SD, standard deviation; SF, Stool Frequency

The median AP score was 0.8 at Weeks 12 and 52 for the PGRS “very mild” category, and 1.0 at Week 12 and 0.8 at Week 52 for the PGIC “much better” category (Table 6, Suppl. Table 8). This estimate was also similar with the supplemental anchors: the median AP score was 1.0 for participants with IBDQ remission at Week 12, for those with an IBDQ item 13 (abdominal pain frequency) response of 6 (“hardly any of the time”) at Week 12, and for those with “slight problems” according to EQ-5D-5 L Pain/Discomfort at Weeks 12 and 52 (Suppl. Tables 9–10).

The CDF and PDF plots, which supplemented anchor-based analyses, showed good separations between PGRS and PGIC categories (Suppl. Figures 1–8). Participants with SF ≤ 3 and AP ≤ 1 were more likely to have “none” to “mild” PGRS and “a little better” to “very much better” improvement on PGIC.

**Fig. 2:**
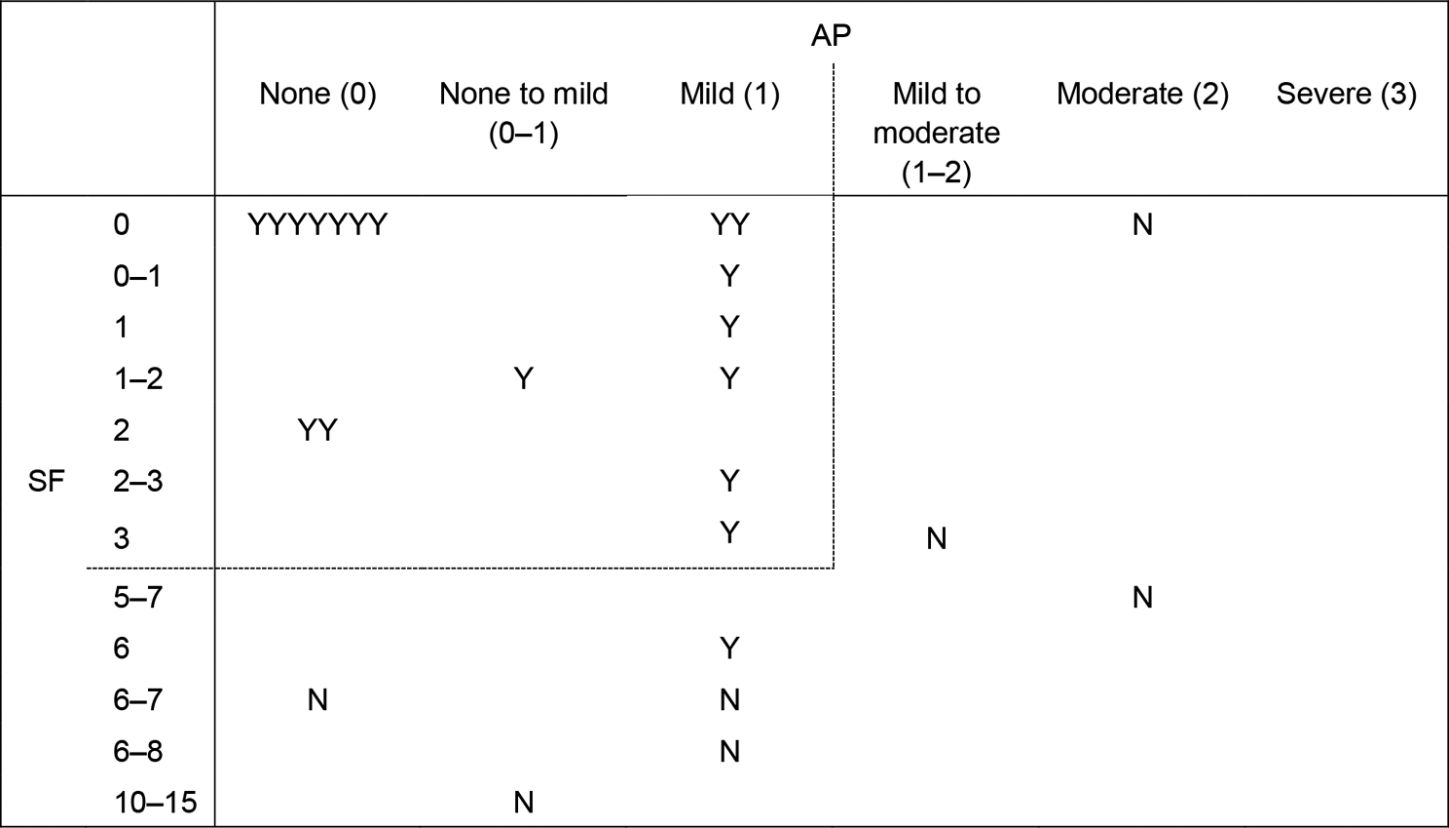
Exit interview responses regarding remission status at the end of VIVID-1. Participants were asked if they considered their Crohn’s disease to be “in remission” at the end of VIVID-1 based on their symptom experience, as assessed by SF (number of Bristol Stool Form Scale type 6 or 7 stools in 24 h) and AP. Each yes (Y) or no (N) response in the table represents one participant. All 17 participants with both SF ≤ 3 and AP ≤ 1 (dashed line) considered themselves to be “in remission”. Abbreviations: AP, Abdominal Pain; SF, Stool Frequency

Exit interviews further assessed this definition of overall clinical remission by PRO; 20 of the 27 interview participants (74%) would consider having ≤ 3 type 6 or 7 bowel movements (SF ≤ 3) and mild/no abdominal pain (AP ≤ 1) in 24 h on most days as being “in remission.” Additionally, all 17 interview participants who reported AP ≤ 1 and SF ≤ 3 considered themselves in remission at the end of the trial (Fig. 2).

## Discussion

Active CD can greatly affect patients’ quality of life [7], and relief of symptoms is an important treatment goal [31]. However, despite their frequency and impact on patients, CD symptoms may be overlooked [32]. This study used data from the Phase 3 VIVID-1 trial of moderately to severely active CD to evaluate the measurement properties of the PRO (SF/AP) items, which assess stool frequency and abdominal pain separately. To our knowledge, this is the first comprehensive study of the psychometric properties of the SF and AP items analyzed as individual items in a large sample, whereas most previous research evaluated the performance of the combined weighted measure (PRO2) [17, 19, 20]. The ability to use these two items separately may better reflect the patient experience than using the full CDAI, and it makes their use more feasible in clinical settings. Our proposed thresholds could be used to inform treatment decisions during the clinical management of CD.

Overall, the SF and AP items of the PRO demonstrated excellent measurement properties to assess stool frequency and abdominal pain in the study population. There were no extreme floor or ceiling effects at study Baseline, Week 12, or Week 52. SF and AP scores were skewed toward the higher (more severe) end of the scale at Baseline, whereas responses were skewed toward the lower (less severe) end of the scale at Week 52, which is consistent with the general trend of improvement in CD symptoms that was observed during the study. In participants defined as stable based on the PGRS and PGIC, the ICCs of the SF and AP score were either close to or above 0.70, indicating good test-retest reliability.

We anticipated that the SF and AP items would correlate strongly with assessments of symptom severity and weakly with endoscopic/laboratory assessments. This was expected based on patient reports typically being more proximal to the patients’ experience than clinical assessments, and it was consistent with extensive evidence from previous IBD studies [15, 33–36]. Hypotheses around construct validity were met for SF (at Weeks 12 and 52) and AP (at all timepoints), supporting the convergent and discriminant validity of both PRO items. These results are consistent with previous research examining the validity of the SF and AP items of the CDAI. In a sample comprising participants with active and inactive CD, the PRO items (SF/AP) showed moderate to large correlations with the IBDQ total score [20]. By contrast, correlations between the PRO items and the SES-CD were weak to moderate in an analysis of pooled data from three other trials of moderately to severely active CD [15].

The present analysis also investigated the known-groups validity and responsiveness of both PRO items, which to our knowledge had not previously been published. Results demonstrated strong known-groups validity for the SF and AP items, as they were able to discriminate between subgroups based on patient-reported measures of global disease severity as well as based on bowel symptoms (for SF) or pain/discomfort (for AP).

The SF and AP components of the PRO were also responsive to change, with moderate to strong correlations with changes in other measures, and statistically significantly greater improvement in SF and AP corresponded to improvement in anchor groups. These findings support the ability of both items to detect change in symptom severity and quality of life among patients with CD as well as to differentiate between levels of change.

The interpretation of the score on a patient-reported outcome measure is important to evaluate treatment benefit, and as such, what constitutes remission needs to be assessed. Here, anchor-based analyses of VIVID-1 data were used to evaluate the threshold that could represent a state of remission by PRO (SF/AP items). For SF, the result supported a threshold in the range of 1–2 points for remission based on PGRS “very mild” and PGIC “much better” improvement. A higher threshold of 3 could indicate mild to moderate symptom severity that participants still recognize as notable symptom improvement, based on PGIC (“a little better”) and PGRS (“mild” or “moderate”). For AP, the estimates supported a threshold of 1 as a state of remission. These findings were supported by CDF/PDF plots.

The exit interviews supported the proposed overall definition of clinical remission by PRO (SF ≤ 3 [per Bristol Stool Form Scale type 6 or 7] and AP ≤ 1, with neither score being worse than Baseline). Therefore, this pre-specified definition, which represents well-controlled abdominal pain but some stool frequency, could represent a state of remission in moderately to severely active CD. This definition is also consistent with previous CD research. While an SF score ≤ 1.5 and an AP score ≤ 1 reflected CDAI-defined remission in an analysis of moderate CD [17], scores ≤ 3 for SF and ≤ 1 for AP have been suggested to better reflect clinical remission in moderately to severely active CD [19]. Further, in clinical trials of moderately to severely active CD, SF scores ≤ 2.8–3 and AP scores ≤ 1 were associated with the greatest treatment effect [18] and correlated with improved general wellbeing (defined by IBDQ item 10 response) [7]. Overall, the current findings support the use of the PRO items to assess stool frequency and abdominal pain severity.

These analyses have several strengths including the large sample size for psychometric evaluation, due to using Phase 3 trial data. Psychometric analyses were conducted in accordance with current standards [37, 38]. Specifically, as recommended by the FDA [39], a variety of instruments were used to evaluate the psychometric properties of the PRO (SF/AP) items, including global instruments (PGRS and PGIC), and disease-specific instruments (e.g., IBDQ item 1 for bowel movement frequency, IBDQ item 13 for abdominal pain frequency). Further, VIVID-1 participants were recruited across multiple countries [40], reflecting varied demographics. Additionally, exit interviews were conducted to provide qualitative evidence to further support the proposed clinical remission thresholds for the PRO items.

These analyses also present limitations. These results may not be generalizable to other patient populations. For instance, different remission thresholds have been previously suggested for patients with less severe CD [17, 19]. Since most participants in this study were White or Asian, the results may not apply to other races. The remission threshold analyses only included patient-reported outcome measures as anchors and did not incorporate objective clinical outcomes; however, given the weak correlations between the PRO and clinical outcomes, inclusion of the latter as anchors would have been inappropriate [41]. Lastly, although a 4-week timeframe has been used previously [42–44], test-retest analyses are often conducted with a shorter timeframe (e.g., 1–2 weeks). The two timepoints should be far enough apart to prevent recall of the prior response, but close enough that no clinically meaningful changes occur [45]. Our test-retest analyses were conducted in subsamples of participants defined as stable using two global assessments (PGRS and PGIC). Future analyses using a shorter interval could further confirm these findings.

## Conclusions

The PRO items (SF/AP) are reliable, construct-valid, and sensitive to change when administered to adults with moderately to severely active CD. Further, anchor-based analyses and exit interviews suggested that a combination of SF ≤ 3 and AP ≤ 1 could represent clinical remission in moderately to severely active CD. Therefore, the PRO items may be used to assess the efficacy of new CD treatments in clinical trial settings but could also be useful during routine clinical management of this condition.

## Electronic supplementary material

Below is the link to the electronic supplementary material.

Supplementary Material 1

## Data Availability

Eli Lilly and Company provides access to all individual participant data collected during the study, after anonymization. Data are available to request 6 months after the indication studied has been approved in the USA and EU and after primary publication acceptability, whichever is later. No expiration date of data requests is currently set once data are made available. Access is provided after a proposal has been approved by an independent review committee identified for this purpose and after receipt of a signed data sharing agreement. Data and documents, including the study protocol, statistical analysis plan, and study report will be provided in a secure data sharing environment. For details on submitting a request, see the instructions provided at [www.vivli.org].

## Abbreviations

ANOVA: Analysis of variance
ANCOVA: Analysis of covariance
AP: Abdominal Pain
CD: Crohn’s disease
CDAI: Crohn’s Disease Activity Index
CDF: Cumulative distribution function
hsCRP: High-sensitivity C-reactive protein
IBD: Inflammatory bowel disease
IBDQ: Inflammatory Bowel Disease Questionnaire
ICC: Intraclass correlation coefficient
PDF: Probability density function
PGIC: Patient Global Impression of Change
PGRS: Patient Global Rating of Severity
PRO: Patient Reported Outcome
SD: Standard deviation
SES-CD: Simple Endoscopic Score for Crohn’s Disease
SF: Stool Frequency
SF-36: 36-Item Short Form Health Survey
