# Supplementary material for: A patient-reported outcome measure comprising the stool frequency and abdominal pain items from the Crohn’s Disease Activity Index: psychometric evaluation in adults with Crohn’s disease
Source: J Patient Rep Outcomes. 2025 Feb 17;9:19. doi: 10.1186/s41687-025-00851-y (PMC11833035; doi:10.1186/s41687-025-00851-y)
Supplement: Supplementary file 1 — Supplementary Material 1 [file 41687_2025_851_MOESM1_ESM.docx]

**A patient-reported outcome measure comprising the stool frequency and abdominal pain items from the Crohn’s Disease Activity Index: psychometric evaluation in adults with Crohn’s disease**

# Supplementary Materials

## Supplementary Table 1. Known-groups validity of the PRO (SF and AP) items by PGRS at Week 12.

| PGRS scores | 1=None | 2=Very mild | 3=Mild | 4=Moderate | 5=Severe | 6=Very severe |
| --- | --- | --- | --- | --- | --- | --- |
| SF scores | N=107 | N=211 | N=274 | N=289 | N=109 | N=26 |
| Mean (SD) | 1.0 (1.6) | 1.8 (1.8) | 2.4 (2.0) | 3.9 (2.7) | 5.8 (4.3) | 6.6 (3.8) |
| Median (range) | 0.3 (0.0, 7.9) | 1.3 (0.0, 10.0) | 2.0 (0.0, 14.0) | 3.7 (0.0, 19.0) | 5.0 (0.0, 26.4) | 6.7 (0.9, 17.9) |
| Effect size |  |  |  |  |  |  |
| vs. 1=None | – | 0.5 | 0.8 | 1.2 | 1.5 | 2.6 |
| vs. 2=Very mild | – | – | 0.3 | 0.9 | 1.4 | 2.3 |
| vs. 3=Mild | – | – | – | 0.6 | 1.2 | 1.9 |
| vs. 4=Moderate | – | – | – | – | 0.6 | 1.0 |
| vs. 5=Severe | – | – | – | – | – | 0.2 |
| LSM (SE) | 1.0 (0.2) | 1.8 (0.2) | 2.4 (0.1) | 3.9 (0.1) | 5.8 (0.2) | 6.6 (0.5) |
| LSMD (CI) |  |  |  |  |  |  |
| vs. 1=None | – | 0.8 (−0.2, 1.8) | 1.5 (0.5, 2.4)*** | 3.0 (2.0, 3.9)**** | 4.8 (3.6, 5.9)**** | 5.6 (3.8, 7.5)**** |
| vs. 2=Very mild | – | – | 0.6 (−0.2, 1.4) | 2.1 (1.3, 2.9)**** | 3.9 (2.9, 4.9)**** | 4.8 (3.0, 6.5)**** |
| vs. 3=Mild | – | – | – | 1.5 (0.8, 2.2)**** | 3.3 (2.4, 4.3)**** | 4.2 (2.4, 5.9)**** |
| vs. 4=Moderate | – | – | – | – | 1.8 (0.9, 2.8)**** | 2.7 (0.9, 4.4)**** |
| vs. 5=Severe | – | – | – | – | – | 0.8 (−1.0, 2.7) |
| AP scores | N=107 | N=211 | N=274 | N=289 | N=109 | N=26 |
| Mean (SD) | 0.1 (0.2) | 0.7 (0.4) | 1.1 (0.3) | 1.7 (0.5) | 2.2 (0.6) | 2.8 (0.4) |
| Median (range) | 0.0 (0.0, 1.1) | 0.8 (0.0, 1.6) | 1.0 (0.0, 2.0) | 2.0 (0.0, 2.7) | 2.1 (0.0, 3.0) | 3.0 (1.0, 3.0) |
| Effect size |  |  |  |  |  |  |
| vs. 1=None | – | 1.6 | 3.1 | 3.5 | 5.1 | 10.4 |
| vs. 2=Very mild | – | – | 1.2 | 2.3 | 3.5 | 5.4 |
| vs. 3=Mild | – | – | – | 1.4 | 2.8 | 4.9 |
| vs. 4=Moderate | – | – | – | – | 1.0 | 2.1 |
| vs. 5=Severe | – | – | – | – | – | 1.0 |
| LSM (SE) | 0.1 (0.0) | 0.7 (0.0) | 1.1 (0.0) | 1.7 (0.0) | 2.2 (0.0) | 2.8 (0.1) |
| LSMD (CI) |  |  |  |  |  |  |
| vs. 1=None | – | 0.5 (0.4, 0.7)**** | 1.0 (0.8, 1.1)**** | 1.6 (1.4, 1.8)**** | 2.1 (1.9, 2.3)**** | 2.7 (2.4, 3.0)**** |
| vs. 2=Very mild | – | – | 0.4 (0.3, 0.5)**** | 1.1 (0.9, 1.2)**** | 1.6 (1.4, 1.8)**** | 2.1 (1.8, 2.4)**** |
| vs. 3=Mild | – | – | – | 0.6 (0.5, 0.8)**** | 1.2 (1.0, 1.3)**** | 1.7 (1.4, 2.0)**** |
| vs. 4=Moderate | – | – | – | – | 0.5 (0.4, 0.7)**** | 1.1 (0.8, 1.4)**** |
| vs. 5=Severe | – | – | – | – | – | 0.6 (0.2, 0.9)**** |

*p <0.05, ** p <0.01, *** p <0.001, **** p <0.0001. Effect size was estimated using Cohen’s d (mean difference divided by the pooled SD). Pairwise LSMD (CI) and p-values were adjusted using Scheffe’s correction.
Abbreviations: AP, Abdominal Pain; CI, confidence interval; LSM, least square mean; LSMD, least square mean difference; PGRS, Patient Global Rating of Severity; PRO, Patient Reported Outcome; SD, standard deviation; SE, standard error; SF, Stool Frequency.

## Supplementary Table 2. Known-groups validity of the PRO (SF and AP) items by PGRS at Week 52.

| PGRS scores | 1=None | 2=Very mild | 3=Mild | 4=Moderate | 5=Severe | 6=Very severe |
| --- | --- | --- | --- | --- | --- | --- |
| SF scores | N=226 | N=234 | N=222 | N=134 | N=41 | N=2 |
| Mean (SD) | 0.7 (1.2) | 1.4 (1.5) | 2.3 (1.8) | 3.0 (2.2) | 5.6 (4.7) | 7.1 (1.2) |
| Median (range) | 0.2 (0.0, 6.3) | 0.9 (0.0, 11.4) | 2.1 (0.0, 10.7) | 2.5 (0.0, 9.0) | 5.0 (0.0, 24.1) | 7.1 (6.3, 8.0) |
| Effect size |  |  |  |  |  |  |
| vs. 1=None | – | 0.5 | 1.0 | 1.4 | 2.3 | 5.4 |
| vs. 2=Very mild | – | – | 0.5 | 0.9 | 1.9 | 3.8 |
| vs. 3=Mild | – | – | – | 0.3 | 1.3 | 2.6 |
| vs. 4=Moderate | – | – | – | – | 0.9 | 1.9 |
| vs. 5=Severe | – | – | – | – | – | 0.3 |
| LSM (SE) | 0.7 (0.1) | 1.4 (0.1) | 2.3 (0.1) | 3.0 (0.2) | 5.6 (0.3) | 7.1 (1.4) |
| LSMD (CI) |  |  |  |  |  |  |
| vs. 1=None | – | 0.7 (0.1, 1.3)* | 1.5 (0.9, 2.2)**** | 2.2 (1.5, 2.9)**** | 4.9 (3.8, 6.0)**** | 6.4 (1.8, 11.0)*** |
| vs. 2=Very mild | – | – | 0.9 (0.3, 1.5)*** | 1.6 (0.9, 2.3)**** | 4.3 (3.2, 5.4)**** | 5.8 (1.2, 10.3)** |
| vs. 3=Mild | – | – | – | 0.7 (−0.0, 1.4) | 3.4 (2.3, 4.5)**** | 4.9 (0.3, 9.4)* |
| vs. 4=Moderate | – | – | – | – | 2.7 (1.5, 3.8)**** | 4.2 (−0.4, 8.8) |
| vs. 5=Severe | – | – | – | – | – | 1.5 (−3.2, 6.2) |
| AP scores | N=226 | N=234 | N=222 | N=134 | N=41 | N=2 |
| Mean (SD) | 0.1 (0.2) | 0.7 (0.4) | 1.0 (0.4) | 1.6 (0.6) | 2.1 (0.7) | 2.0 (1.4) |
| Median (range) | 0.0 (0.0, 1.3) | 0.8 (0.0, 1.6) | 1.0 (0.0, 3.0) | 1.9 (0.0, 2.6) | 2.1 (0.0, 3.0) | 2.0 (1.0, 3.0) |
| Effect size |  |  |  |  |  |  |
| vs. 1=None | – | 1.9 | 3.0 | 3.7 | 6.2 | 10.4 |
| vs. 2=Very mild | – | – | 0.8 | 1.8 | 3.0 | 3.2 |
| vs. 3=Mild | – | – | – | 1.2 | 2.3 | 2.4 |
| vs. 4=Moderate | – | – | – | – | 0.7 | 0.7 |
| vs. 5=Severe | – | – | – | – | – | –0.1 |
| LSM (SE) | 0.1 (0.0) | 0.7 (0.0) | 1.0 (0.0) | 1.6 (0.0) | 2.1 (0.1) | 2.0 (0.3) |
| LSMD (CI) |  |  |  |  |  |  |
| vs. 1=None | – | 0.6 (0.4, 0.7)**** | 0.9 (0.8, 1.0)**** | 1.5 (1.3, 1.7)**** | 2.0 (1.7, 2.2)**** | 1.9 (0.9, 2.9)**** |
| vs. 2=Very mild | – | – | 0.3 (0.2, 0.5)**** | 0.9 (0.8, 1.1)**** | 1.4 (1.2, 1.6)**** | 1.3 (0.3, 2.4)** |
| vs. 3=Mild | – | – | – | 0.6 (0.4, 0.7)**** | 1.1 (0.8, 1.3)**** | 1.0 (−0.0, 2.0) |
| vs. 4=Moderate | – | – | – | – | 0.5 (0.2, 0.7)**** | 0.4 (−0.6, 1.4) |
| vs. 5=Severe | – | – | – | – | – | −0.1 (−1.1, 1.0) |

*p <0.05, ** p <0.01, *** p <0.001, **** p <0.0001. Effect size was estimated using Cohen’s d (mean difference divided by the pooled SD). Pairwise LSMD (CI) and p-values were adjusted using Scheffe’s correction.
Abbreviations: AP, Abdominal Pain; CI, confidence interval; LSM, least square mean; LSMD, least square mean difference; PGRS, Patient Global Rating of Severity; PRO, Patient Reported Outcome; SD, standard deviation; SE, standard error; SF, Stool Frequency.

## Supplementary Table 3. Known-groups validity of the PRO AP item by EQ-5D-5L Pain/Discomfort groups.

| EQ-5D-5L Pain/Discomfort group scores | 1=None | 2=Slight | 3=Moderate | 4=Severe | 5=Extreme |
| --- | --- | --- | --- | --- | --- |
| AP scores at Baseline | N=64 | N=247 | N=455 | N=256 | N=37 |
| Mean (SD) | 1.7 (0.9) | 1.8 (0.6) | 2.1 (0.4) | 2.5 (0.5) | 2.7 (0.4) |
| Median (range) | 2.0 (0.0, 3.0) | 2.0 (0.0, 3.0) | 2.0 (0.3, 3.0) | 2.6 (0.3, 3.0) | 2.9 (1.3, 3.0) |
| Effect size |  |  |  |  |  |
| vs. 1=None | – | 0.3 | 0.8 | 1.4 | 1.4 |
| vs. 2=Slight | – | – | 0.5 | 1.2 | 1.4 |
| vs. 3=Moderate | – | – | – | 0.9 | 1.3 |
| vs. 4=Severe | – | – | – | – | 0.5 |
| LSM (SE) | 1.7 (0.1) | 1.8 (0.0) | 2.1 (0.0) | 2.5 (0.0) | 2.7 (0.1) |
| LSMD (CI) |  |  |  |  |  |
| vs. 1=None | – | 0.2 (−0.1, 0.4) | 0.4 (0.2, 0.6)**** | 0.8 (0.6, 1.0)**** | 1.0 (0.7, 1.3)**** |
| vs. 2=Slight | – | – | 0.2 (0.1, 0.4)**** | 0.6 (0.5, 0.8)**** | 0.8 (0.6, 1.1)**** |
| vs. 3=Moderate | – | – | – | 0.4 (0.3, 0.5)**** | 0.6 (0.3, 0.9)**** |
| vs. 4=Severe | – | – | – | – | 0.2 (−0.1, 0.5) |
| AP scores at Week 12 | N=262 | N=438 | N=240 | N=62 | N=7 |
| Mean (SD) | 0.7 (0.7) | 1.2 (0.7) | 1.7 (0.6) | 2.2 (0.7) | 2.2 (1.0) |
| Median (range) | 0.6 (0.0, 3.0) | 1.0 (0.0, 3.0) | 1.9 (0.0, 3.0) | 2.2 (0.0, 3.0) | 2.6 (0.1, 3.0) |
| Effect size |  |  |  |  |  |
| vs. 1=None | – | 0.7 | 1.5 | 2.1 | 2.1 |
| vs. 2=Slight | – | – | 0.8 | 1.5 | 1.5 |
| vs. 3=Moderate | – | – | – | 0.8 | 0.9 |
| vs. 4=Severe | – | – | – | – | 0.0 |
| LSM (SE) | 0.7 (0.0) | 1.2 (0.0) | 1.7 (0.0) | 2.2 (0.1) | 2.2 (0.2) |
| LSMD (CI) |  |  |  |  |  |
| vs. 1=None | – | 0.5 (0.3, 0.7)**** | 1.0 (0.8, 1.2)**** | 1.5 (1.2, 1.8)**** | 1.5 (0.7, 2.3)**** |
| vs. 2=Slight | – | – | 0.5 (0.3, 0.6)**** | 1.0 (0.7, 1.3)**** | 1.0 (0.2, 1.8)** |
| vs. 3=Moderate | – | – | – | 0.5 (0.2, 0.8)**** | 0.5 (−0.3, 1.3) |
| vs. 4=Severe | – | – | – | – | 0.0 (−0.8, 0.8) |
| AP scores at Week 52 | N=378 | N=325 | N=120 | N=9 | N=3 |
| Mean (SD) | 0.5 (0.6) | 0.9 (0.6) | 1.5 (0.7) | 1.5 (0.7) | 0.8 (0.7) |
| Median (range) | 0.1 (0.0, 3.0) | 1.0 (0.0, 3.0) | 1.7 (0.0, 3.0) | 1.9 (0.0, 2.3) | 0.7 (0.1, 1.6) |
| Effect size |  |  |  |  |  |
| vs. 1=None | – | 0.7 | 1.6 | 1.7 | 0.5 |
| vs. 2=Slight | – | – | 1.0 | 1.1 | −0.1 |
| vs. 3=Moderate | – | – | – | 0.0 | −1.0 |
| vs. 4=Severe | – | – | – | – | −1.0 |
| LSM (SE) | 0.5 (0.0) | 0.9 (0.0) | 1.5 (0.1) | 1.5 (0.2) | 0.8 (0.4) |
| LSMD (CI) |  |  |  |  |  |
| vs. 1=None | – | 0.4 (0.3, 0.6)**** | 1.0 (0.8, 1.2)**** | 1.1 (0.4, 1.7)**** | 0.3 (−0.8, 1.4) |
| vs. 2=Slight | – | – | 0.6 (0.4, 0.8)**** | 0.6 (0.0, 1.3)* | −0.1 (−1.2, 1.0) |
| vs. 3=Moderate | – | – | – | 0.0 (−0.6, 0.7) | −0.7 (−1.8, 0.4) |
| vs. 4=Severe | – | – | – | – | −0.7 (−2.0, 0.5) |

*p <0.05, ** p <0.01, *** p <0.001, **** p <0.0001. Effect size was estimated using Cohen’s d (mean difference divided by the pooled SD). Pairwise LSMD (CI) and p-values were adjusted using Scheffe’s correction.
Abbreviations: AP, Abdominal Pain; CI, confidence interval; LSM, least square mean; LSMD, least square mean difference; PRO, Patient Reported Outcome; SD, standard deviation; SE, standard error.

## Supplementary Table 4. Score change correlations of the PRO (SF and AP) items with anchor measures.

|  | Baseline to Week 12 | | Baseline to Week 52 | |
| --- | --- | --- | --- | --- |
|  | N | Spearman ρ (95% CI) | N | Spearman ρ (95% CI) |
| Score change correlations with SF |  |  |  |  |
| PGRS average change | 1011 | 0.52 (0.47, 0.56) | 855 | 0.40 (0.34, 0.45) |
| PGIC | 1005 | 0.45 (0.40, 0.49) | 831 | 0.32 (0.26, 0.38) |
| IBDQ Bowel Symptom domain score change | 1001 | −0.46 (−0.51, −0.41) | 830 | −0.35 (−0.41, −0.29) |
| IBDQ item 1 (bowel movement frequency) score change | 1001 | −0.43 (−0.48, −0.38) | 830 | −0.38 (−0.43, −0.32) |
| IBDQ total score change | 1001 | −0.42 (−0.47, −0.37) | 830 | −0.31 (−0.37, −0.25) |
| Score change correlations with AP |  |  |  |  |
| PGRS average change | 1013 | 0.85 (0.84, 0.87) | 858 | 0.82 (0.79, 0.84) |
| PGIC | 1007 | 0.47 (0.42, 0.52) | 834 | 0.36 (0.30, 0.42) |
| IBDQ item 13 (abdominal pain frequency) score change | 1003 | −0.51 (−0.55, −0.46) | 833 | −0.47 (−0.52, −0.42) |
| IBDQ total score change | 1003 | −0.49 (−0.54, −0.44) | 833 | −0.40 (−0.45, −0.34) |

Abbreviations: AP, abdominal pain; CI, confidence interval; IBDQ, Inflammatory Bowel Disease Questionnaire; PGIC, Patient Global Impression of Change; PGRS, Patient Global Rating of Severity; PRO, Patient Reported Outcome; SF, stool frequency.

## Supplementary Table 5. Responsiveness of the PRO (SF and AP) items by PGIC at Weeks 12 and 52.

| PGIC categories | Very much better | Much better | A little better | No change | A little worse | Much worse | Very much worse |
| --- | --- | --- | --- | --- | --- | --- | --- |
| SF score change from Baseline to Week 12 | N=153 | N=323 | N=308 | N=159 | N=33 | N=21 | N=8 |
| Mean (SD) | −4.4 (2.9) | −3.5 (2.6) | −2.1 (2.3) | −1.4 (2.2) | −0.8 (2.0) | −0.7 (2.0) | 2.2 (6.0) |
| Median (range) | −4.1 (−14.2, 0.6) | −3.4 (−17.9, 3.7) | −1.9 (−10.9, 11.9) | −0.7 (−10.6, 5.1) | −0.6 (−6.4, 4.3) | −0.6 (−4.3, 4.1) | 1.0 (−4.4, 16.0) |
| Effect size |  |  |  |  |  |  |  |
| vs. very much better | – | –0.3 | –0.9 | –1.2 | –1.3 | –1.3 | –2.2 |
| vs. much better | – | – | –0.6 | –0.9 | –1.1 | –1.1 | –2.1 |
| vs. a little better | – | – | – | –0.3 | –0.6 | –0.6 | –1.8 |
| vs. no change | – | – | – | – | –0.3 | –0.3 | –1.4 |
| vs. a little worse | – | – | – | – | – | 0.0 | –1.0 |
| vs. much worse | – | – | – | – | – | – | –0.8 |
| LSM (SE) | −4.4 (0.2) | −3.6 (0.1) | −2.1 (0.1) | −1.2 (0.2) | −0.6 (0.4) | −0.6 (0.4) | 2.6 (0.7) |
| LSMD (CI) |  |  |  |  |  |  |  |
| vs. very much better | – | −0.8 (−1.5, −0.0) | −2.2 (−2.9, −1.5) | −3.2 (−4.0, −2.3) | −3.7 (−5.1, −2.3) | −3.8 (−5.5, −2.0) | −6.9 (−9.6, −4.2) |
| vs. much better | – | – | −1.4 (−2.0, −0.9) | −2.4 (−3.1, −1.7) | −2.9 (−4.3, −1.6) | −3.0 (−4.7, −1.3) | −6.2 (−8.8, −3.5) |
| vs. a little better | – | – | – | −0.9 (−1.7, −0.2) | −1.5 (−2.8, −0.1) | −1.5   (−3.2, 0.1) | −4.7 (−7.4, −2.1) |
| vs. no change | – | – | – | – | −0.6 (−2.0, 0.9) | −0.6   (−2.3, 1.1) | −3.8 (−6.4, −1.1) |
| vs. a little worse | – | – | – | – | – | −0.0   (−2.1, 2.0) | −3.2 (−6.1, −0.3) |
| vs. much worse | – | – | – | – | – | – | −3.2 (−6.2, −0.1) |
| SF score change from Baseline to Week 52 | N=278 | N=348 | N=146 | N=40 | N=12 | N=3 | N=4 |
| Mean (SD) | −4.8 (3.1) | −3.7 (2.7) | −2.6 (2.3) | −2.5 (2.7) | −1.0 (1.9) | 0.9 (2.3) | −3.3 (1.7) |
| Median (range) | −4.6 (−20.9, 2.4) | −3.6 (−19.6, 7.3) | −2.1 (−11.1, 3.7) | −2.3 (−12.0, 3.0) | −1.4 (−3.3, 3.4) | 1.9 (−1.7, 2.6) | −2.6 (−5.9, −2.3) |
| Effect size |  |  |  |  |  |  |  |
| vs. very much better | – | –0.4 | –0.8 | –0.7 | –1.2 | –1.8 | –0.5 |
| vs. much better | – | – | –0.4 | –0.4 | –1.0 | –1.7 | –0.1 |
| vs. a little better | – | – | – | 0.0 | –0.7 | –1.5 | 0.3 |
| vs. no change | – | – | – | – | –0.6 | –1.3 | 0.3 |
| vs. a little worse | – | – | – | – | – | –1.0 | 1.2 |
| vs. much worse | – | – | – | – | – | – | 2.2 |
| LSM (SE) | −4.7 (0.1) | −3.8 (0.1) | −2.6 (0.2) | −2.1 (0.3) | −1.3 (0.5) | 1.1 (1.1) | −3.5 (0.9) |
| LSMD (CI) |  |  |  |  |  |  |  |
| vs. very much better | – | −0.9 (−1.4, −0.3) | −2.1 (−2.7, −1.4) | −2.6 (−3.8, −1.5) | −3.4 (−5.4, −1.42) | −5.8 (−9.7, −1.9) | −1.2   (−4.6, 2.2) |
| vs. much better | – | – | −1.2 (−1.9, −0.5) | −1.8 (−2.9, −0.6) | −2.5 (−4.5, −0.6) | −4.9 (−8.8, −1.0) | −0.4 (−3.7, 3.0) |
| vs. a little better | – | – | – | −0.6   (−1.8, 0.6) | −1.3   (−3.4, 0.7) | −3.7   (−7.6, 0.2) | 0.8 (−2.6, 4.2) |
| vs. no change | – | – | – | – | −0.8   (−3.0, 1.4) | −3.1   (−7.1, 0.9) | 1.4 (−2.1, 4.9) |
| vs. a little worse | – | – | – | – | – | −2.4   (−6.7, 2.0) | 2.2 (−1.7, 6.1) |
| vs. much worse | – | – | – | – | – | – | 4.5 (−0.6, 9.7) |
| AP score change from Baseline to Week 12 | N=154 | N=325 | N=308 | N=159 | N=33 | N=20 | N=8 |
| Mean (SD) | −1.5 (0.7) | −1.1 (0.7) | −0.7 (0.7) | −0.4 (0.7) | −0.3 (0.6) | −0.4 (0.7) | 0.0 (0.6) |
| Median (range) | −1.6 (−3.0, 0.3) | −1.0 (−3.0, 0.7) | −0.6 (−2.7, 1.4) | −0.1 (−2.9, 1.0) | −0.4 (−1.9, 1.0) | −0.2 (−1.9, 1.0) | 0.1 (−1.0, 0.9) |
| Effect size |  |  |  |  |  |  |  |
| vs. very much better | – | –0.6 | –1.2 | –1.5 | –1.6 | –1.6 | –2.1 |
| vs. much better | – | – | –0.6 | –0.9 | –1.1 | –1.0 | –1.6 |
| vs. a little better | – | – | – | –0.3 | –0.5 | –0.5 | –1.0 |
| vs. no change | – | – | – | – | –0.2 | –0.1 | –0.7 |
| vs. a little worse | – | – | – | – | – | 0.0 | –0.6 |
| vs. much worse | – | – | – | – | – | – | –0.6 |
| LSM (SE) | −1.5 (0.0) | −1.1 (0.0) | −0.7 (0.0) | −0.4 (0.0) | −0.3 (0.1) | −0.2 (0.1) | 0.0 (0.2) |
| LSMD (CI) |  |  |  |  |  |  |  |
| vs. very much better | – | −0.4 (−0.6, −0.2) | −0.8 (−1.1, −0.6) | −1.1 (−1.3, −0.8) | −1.3 (−1.7, −0.8) | −1.3 (−1.8, −0.7) | −1.5 (−2.3, −0.7) |
| vs. much better | – | – | −0.4 (−0.6, −0.2) | −0.7 (−0.9, −0.5) | −0.8 (−1.2, −0.4) | −0.9 (−1.4, −0.3) | −1.1 (−1.9,−0.3) |
| vs. a little better | – | – | – | −0.3 (−0.5, −0.0) | −0.4 (−0.8, −0.0) | −0.4   (−1.0, 0.1) | −0.7   (−1.5, 0.1) |
| vs. no change | – | – | – | – | −0.2   (−0.6, 0.3) | −0.2   (−0.7, 0.3) | −0.4   (−1.2, 0.4) |
| vs. a little worse | – | – | – | – | – | −0.0   (−0.6, 0.6) | −0.3   (−1.2, 0.6) |
| vs. much worse | – | – | – | – | – | – | −0.3   (−1.2, 0.7) |
| AP score change from Baseline to Week 52 | N=280 | N=349 | N=146 | N=40 | N=12 | N=3 | N=4 |
| Mean (SD) | −1.6 (0.8) | −1.3 (0.8) | −0.9 (0.8) | −0.8 (0.8) | −0.7 (0.6) | −0.5 (0.8) | −1.2 (0.6) |
| Median (range) | −1.9 (−3.0, 1.4) | −1.1 (−3.0, 0.4) | −0.9 (−3.0, 1.0) | −0.7 (−2.3, 0.9) | −0.5 (−2.1, 0.1) | −0.3 (−1.4, 0.1) | −1.3 (−1.9, −0.4) |
| Effect size |  |  |  |  |  |  |  |
| vs. very much better | – | –0.5 | –1.0 | –1.2 | –1.3 | –1.5 | –0.6 |
| vs. much better | – | – | –0.5 | –0.7 | –0.8 | –1.0 | –0.1 |
| vs. a little better | – | – | – | –0.2 | –0.3 | –0.5 | 0.4 |
| vs. no change | – | – | – | – | –0.1 | –0.3 | 0.6 |
| vs. a little worse | – | – | – | – | – | –0.2 | 0.9 |
| vs. much worse | – | – | – | – | – | – | 1.0 |
| LSM (SE) | −1.6 (0.0) | −1.3 (0.0) | −0.9 (0.0) | −0.8 (0.1) | −0.9 (0.2) | −0.3 (0.4) | −1.2 (0.3) |
| LSMD (CI) |  |  |  |  |  |  |  |
| vs. very much better | – | −0.4 (−0.5, −0.2) | −0.7 (−0.9, −0.5) | −0.8 (−1.2, −0.4) | −0.8   (−1.4, −0.1) | −1.3  (−2.6, 0.0) | −0.4  (−1.6, 0.7) |
| vs. much better | – | – | −0.4 (−0.6, −0.1) | −0.4 (−0.8, −0.1) | −0.4 (−1.1, 0.3) | −1.0 (−2.3, 0.4) | −0.1 (−1.2, 1.1) |
| vs. a little better | – | – | – | −0.1   (−0.5, 0.3) | −0.0   (−0.7, 0.6) | −0.6   (−1.9, 0.7) | 0.3 (−0.9, 1.4) |
| vs. no change | – | – | – | – | 0.1 (−0.7, 0.8) | −0.5   (−1.9, 0.8) | 0.4 (−0.8, 1.6) |
| vs. a little worse | – | – | – | – | – | −0.6   (−2.0, 0.9) | 0.3 (−1.0, 1.6) |
| vs. much worse | – | – | – | – | – | – | 0.9  (−0.8, 2.6) |

Effect size was estimated using Cohen’s d (mean difference divided by the pooled SD). Pairwise LSMD (CI) were adjusted using Scheffe’s correction.
Abbreviations: AP, Abdominal Pain; CI, confidence interval; LSM, least square mean; LSMD, least square mean difference; PGIC, Patient Global Impression of Change; PRO, Patient Reported Outcome; SD, standard deviation; SE, standard error; SF, Stool Frequency.

## Supplementary Table 6. Responsiveness of the PRO SF item by IBDQ at Weeks 12 and 52.

|  | IBDQ Bowel Symptom domain response | | IBDQ item 1 (bowel movement frequency) change score | | IBDQ response | | IBDQ remission | |
| --- | --- | --- | --- | --- | --- | --- | --- | --- |
|  | Yes: ≥8-point increase | No: <8-point increase | ≥1-point increase | <1-point increase | Yes: ≥16-point increase | No:  <16-point increase | Yes: ≥170 | No: <170 |
| SF score change from Baseline to Week 12 | N=671 | N=330 | N=743 | N=258 | N=703 | N=298 | N=493 | N=512 |
| Mean (SD) | −3.3 (2.7) | −1.3 (2.4) | −3.1 (2.7) | −1.3 (2.4) | −3.2 (2.7) | −1.3 (2.5) | −3.3 (2.6) | −2.1 (2.8) |
| Median (range) | −3.0 (−17.9, 3.7) | −1.0 (−10.3, 16.0) | −2.9 (−17.9, 11.9) | −0.9 (−10.3, 16.0) | −3.0 (−17.9, 4.3) | −1.0 (−10.3, 16.0) | −3.1 (−14.6, 3.7) | −1.6 (−17.9, 16.0) |
| Effect size |  | –0.8 |  | –0.7 |  | –0.7 |  | –0.5 |
| LSM (SE) | −3.3 (0.1) | −1.3 (0.1) | −3.1 (0.1) | −1.4 (0.1) | −3.3 (0.1) | −1.3 (0.1) | −3.5 (0.1) | −1.9 (0.1) |
| LSMD (CI) |  | −2.0 (−2.3, −1.7) |  | −1.7 (−2.1, −1.4) |  | −2.0 (−2.3, −1.6) |  | −1.6 (−1.9, −1.3) |
| SF score change from Baseline to Week 52 | N=679 | N=151 | N=682 | N=148 | N=695 | N=135 | N=562 | N=270 |
| Mean (SD) | −4.1 (2.9) | −2.3 (2.3) | −4.0 (2.9) | −2.4 (2.5) | −4.0 (2.9) | −2.4 (2.3) | −4.1 (2.8) | −3.0 (3.0) |
| Median (range) | −4.0 (−20.9, 7.3) | −2.0  (−12.9, 2.1) | −3.9  (−20.9, 7.3) | −2.2  (−12.9, 3.0) | −3.9  (−20.9, 7.3) | −2.1  (−12.0, 3.7) | −4.1  (−20.9, 2.4) | −2.6  (−19.6, 7.3) |
| Effect size |  | –0.6 |  | –0.6 |  | –0.6 |  | –0.4 |
| LSM (SE) | −4.1 (0.1) | −2.4 (0.2) | −4.0 (0.1) | −2.7 (0.2) | −4.0 (0.1) | −2.4 (0.2) | −4.3 (0.1) | −2.7 (0.1) |
| LSMD (CI) |  | −1.6 (−2.0, −1.3) |  | −1.2 (−1.6, −0.9) |  | −1.6 (−1.9, −1.2) |  | −1.6 (−1.9, −1.3) |

Effect size was estimated using Cohen’s d (mean difference divided by the pooled SD).

Abbreviations: CI, confidence interval; IBDQ, Inflammatory Bowel Disease Questionnaire; LSM, least square mean; LSMD, least square mean difference; PRO, Patient Reported Outcome; SD, standard deviation; SE, standard error; SF, Stool Frequency.

## Supplementary Table 7. Responsiveness of the PRO AP item by IBDQ at Weeks 12 and 52.

|  | IBDQ item 13 (abdominal pain frequency) change score | | IBDQ response | | IBDQ remission | |
| --- | --- | --- | --- | --- | --- | --- |
|  | ≥1-point increase | <1-point increase | Yes: ≥16-point increase | No: <16-point increase | Yes: ≥170 | No: <170 |
| AP score change from Baseline to Week 12 | N=770 | N=233 | N=703 | N=300 | N=495 | N=512 |
| Mean (SD) | −1.0 (0.8) | −0.4 (0.7) | −1.1 (0.8) | −0.4 (0.6) | −1.2 (0.8) | −0.6 (0.7) |
| Median (range) | −1.0 (−3.0, 1.4) | −0.1 (−2.0, 1.0) | −1.0 (−3.0, 1.1) | −0.3 (−2.4, 1.4) | −1.0 (−3.0, 0.6) | −0.6 (−2.9, 1.4) |
| Effect size | –0.9 | –0.9 | –0.8 | Effect size | –0.9 | –0.9 |
| LSM (SE) | −1.0 (0.0) | −0.4 (0.0) | −1.1 (0.0) | −0.5 (0.0) | −1.2 (0.0) | −0.6 (0.0) |
| LSMD (CI) | −0.6 (−0.7, −0.5) | | −0.6 (−0.7, −0.5) | | −0.7 (−0.7, −0.6) | |
| AP score change from Baseline to Week 52 | N=721 | N=112 | N=696 | N=137 | N=564 | N=271 |
| Mean (SD) | −1.4 (0.8) | −0.7 (0.7) | −1.4 (0.8) | −0.7 (0.7) | −1.5 (0.8) | −1.0 (0.8) |
| Median (range) | −1.4 (−3.0, 0.6) | −0.7 (−2.1, 1.4) | −1.4 (−3.0, 0.6) | −0.7 (−2.3, 1.4) | −1.5 (−3.0, 0.6) | −1.0 (−3.0, 1.4) |
| Effect size | –0.8 | –0.9 | –0.6 | Effect size | –0.8 | –0.9 |
| LSM (SE) | −1.4 (0.0) | −1.0 (0.1) | −1.4 (0.0) | −0.9 (0.1) | −1.5 (0.0) | −0.9 (0.0) |
| LSMD (CI) | −0.4 (−0.5, −0.3) | | −0.5 (−0.6, −0.3) | | −0.6 (−0.7, −0.5) | |

Effect size was estimated using Cohen’s d (mean difference divided by the pooled SD).

Abbreviations: AP, Abdominal Pain; CI, confidence interval; IBDQ, Inflammatory Bowel Disease Questionnaire; LSM, least square mean; LSMD, least square mean difference; PRO, Patient Reported Outcome; SD, standard deviation; SE, standard error.

## Supplementary Table 8. Anchor-based estimates for the PRO (SF and AP) items by PGRS and PGIC at Week 52.

| Anchors | SF item | | | | | | | AP item | | | | | | |
| --- | --- | --- | --- | --- | --- | --- | --- | --- | --- | --- | --- | --- | --- | --- |
|  | N | Mean (SD) | Percentile | | | | | N | Mean (SD) | Percentile | | | | |
|  |  |  | 10^th^ | 25^th^ (Q1) | 50^th^ (Q2) | 75^th^  (Q3) | 90^th^ |  |  | 10^th^ | 25^th^ (Q1) | 50^th^ (Q2) | 75^th^  (Q3) | 90^th^ |
| PGRS |  |  |  |  |  |  |  |  |  |  |  |  |  |  |
| 1=None | 226 | 0.7 (1.2) | 0.0 | 0.0 | 0.2 | 0.9 | 2.1 | 226 | 0.1 (0.2) | 0.0 | 0.0 | 0.0 | 0.1 | 0.3 |
| 2=Very mild | 234 | 1.4 (1.5) | 0.0 | 0.2 | 0.9 | 2.1 | 3.6 | 234 | 0.7 (0.4) | 0.0 | 0.3 | 0.8 | 1.0 | 1.0 |
| 3=Mild | 222 | 2.3 (1.8) | 0.0 | 0.8 | 2.1 | 3.3 | 4.7 | 222 | 1.0 (0.4) | 0.3 | 1.0 | 1.0 | 1.1 | 1.4 |
| 4=Moderate | 134 | 3.0 (2.2) | 0.1 | 1.1 | 2.5 | 4.3 | 6.1 | 134 | 1.6 (0.6) | 0.4 | 1.4 | 1.9 | 2.0 | 2.0 |
| 5=Severe | 41 | 5.6 (4.7) | 1.0 | 2.9 | 5.0 | 7.0 | 10.8 | 41 | 2.1 (0.7) | 1.0 | 2.0 | 2.1 | 2.5 | 2.6 |
| 6=Very Severe | 2 | 7.1 (1.2) | 6.3 | 6.3 | 7.1 | 8.0 | 8.0 | 2 | 2.0 (1.4) | 1.0 | 1.0 | 2.0 | 3.0 | 3.0 |
| PGIC |  |  |  |  |  |  |  |  |  |  |  |  |  |  |
| 1=Very much better | 280 | 1.0 (1.4) | 0.0 | 0.0 | 0.4 | 1.6 | 2.9 | 280 | 0.5 (0.6) | 0.0 | 0.0 | 0.1 | 1.0 | 1.1 |
| 2=Much better | 349 | 1.8 (1.9) | 0.0 | 0.3 | 1.3 | 2.7 | 4.3 | 349 | 0.8 (0.6) | 0.0 | 0.1 | 1.0 | 1.1 | 1.7 |
| 3=A little better | 146 | 3.0 (2.9) | 0.3 | 0.9 | 2.4 | 4.0 | 6.4 | 146 | 1.2 (0.7) | 0.0 | 0.7 | 1.1 | 2.0 | 2.0 |
| 4=No change | 40 | 3.8 (3.3) | 0.1 | 1.4 | 3.5 | 5.2 | 7.2 | 40 | 1.2 (0.8) | 0.0 | 1.0 | 1.0 | 2.0 | 2.2 |
| 5=A little worse | 12 | 4.3 (2.5) | 2.0 | 3.2 | 3.6 | 4.6 | 6.9 | 12 | 1.1 (0.8) | 0.0 | 0.5 | 1.0 | 1.9 | 2.0 |
| 6=Much worse | 3 | 6.8 (1.9) | 5.0 | 5.0 | 6.6 | 8.9 | 8.9 | 3 | 1.9 (0.7) | 1.0 | 1.0 | 2.3 | 2.3 | 2.3 |
| 7=Very much worse | 4 | 2.1 (3.3) | 0.0 | 0.1 | 0.8 | 4.2 | 7.0 | 4 | 0.9 (0.6) | 0.1 | 0.4 | 0.9 | 1.4 | 1.6 |

Abbreviations: AP, Abdominal Pain; PGRS, Patient Global Rating of Severity; PGIC, Patient Global Impression of Change; PRO, Patient Reported Outcome; Q, quartile; SD, standard deviation; SF, Stool Frequency.

## Supplementary Table 9. Anchor-based estimates for the PRO (SF and AP) items by supplemental anchors at Week 12.

| Anchors | SF item | | | | | | | AP item | | | | | | |
| --- | --- | --- | --- | --- | --- | --- | --- | --- | --- | --- | --- | --- | --- | --- |
|  | N | Mean (SD) | Percentile | | | | | N | Mean (SD) | Percentile | | | | |
|  |  |  | 10^th^ | 25^th^ (Q1) | 50^th^ (Q2) | 75^th^  (Q3) | 90^th^ |  |  | 10^th^ | 25^th^ (Q1) | 50^th^ (Q2) | 75^th^  (Q3) | 90^th^ |
| IBDQ remission^a^ |  |  |  |  |  |  |  |  |  |  |  |  |  |  |
| Yes: ≥170 | 495 | 2.0 (2.0) | 0.0 | 0.4 | 1.4 | 3.1 | 5.0 | 495 | 0.9 (0.7) | 0.0 | 0.1 | 1.0 | 1.1 | 2.0 |
| No: <170 | 514 | 4.1 (3.4) | 0.6 | 1.6 | 3.7 | 5.7 | 7.3 | 514 | 1.6 (0.7) | 0.7 | 1.0 | 1.7 | 2.0 | 2.6 |
| IBDQ Bowel Symptom domain responseᵇ |  |  |  |  |  |  |  |  |  |  |  |  |  |  |
| ≥8-point increase | 672 | 2.4 (2.4) | 0.0 | 0.6 | 1.7 | 3.8 | 5.9 | – | – | – | – | – | – | – |
| <8-point increase | 333 | 4.4 (3.5) | 0.7 | 2.1 | 4.0 | 5.9 | 7.4 | – | – | – | – | – | – | – |
| IBDQ item 1 (bowel movement frequency) |  |  |  |  |  |  |  | – | – | – | – | – | – | – |
| 7=Normal, no increase in frequency of bowel movements | 447 | 2.0 (2.0) | 0.0 | 0.3 | 1.3 | 3.1 | 5.0 | – | – | – | – | – | – | – |
| 6=Slight increase in frequency of bowel movements | 166 | 2.5 (2.4) | 0.1 | 0.9 | 1.9 | 3.6 | 5.4 | – | – | – | – | – | – | – |
| 5=Some increase in frequency of bowel movements | 99 | 3.0 (2.1) | 0.3 | 1.3 | 3.0 | 4.3 | 5.9 | – | – | – | – | – | – | – |
| 4=Moderate increase in frequency of bowel movements | 108 | 3.7 (2.6) | 0.7 | 1.9 | 3.1 | 5.3 | 6.9 | – | – | – | – | – | – | – |
| 3=Very frequent | 122 | 5.5 (3.5) | 1.7 | 3.9 | 5.0 | 6.7 | 8.3 | – | – | – | – | – | – | – |
| 2=Extremely frequent | 46 | 7.0 (4.2) | 2.7 | 4.1 | 6.0 | 8.6 | 13.6 | – | – | – | – | – | – | – |
| 1=Bowel movements as or more frequent than they have ever been | 21 | 5.6 (4.3) | 2.1 | 3.2 | 4.6 | 6.6 | 8.4 | – | – | – | – | – | – | – |
| IBDQ item 13 (abdominal pain frequency) |  |  |  |  |  |  |  |  |  |  |  |  |  |  |
| 7=None of the time | – | – | – | – | – | – | – | 108 | 0.4 (0.7) | 0.0 | 0.0 | 0.0 | 0.9 | 1.4 |
| 6=Hardly any of the time | – | – | – | – | – | – | – | 252 | 0.8 (0.6) | 0.0 | 0.1 | 1.0 | 1.0 | 1.5 |
| 5=A little of the time | – | – | – | – | – | – | – | 226 | 1.2 (0.6) | 0.3 | 0.9 | 1.0 | 1.7 | 2.0 |
| 4=Some of the time | – | – | – | – | – | – | – | 227 | 1.5 (0.6) | 0.9 | 1.0 | 1.6 | 2.0 | 2.1 |
| 3=A good bit of the time | – | – | – | – | – | – | – | 98 | 1.8 (0.6) | 1.0 | 1.3 | 2.0 | 2.0 | 2.6 |
| 2=Most of the time | – | – | – | – | – | – | – | 62 | 2.0 (0.6) | 1.3 | 1.9 | 2.0 | 2.4 | 3.0 |
| 1=All of the time | – | – | – | – | – | – | – | 36 | 2.3 (0.7) | 1.6 | 1.9 | 2.3 | 2.9 | 3.0 |
| EQ-5D-5L Pain/Discomfort |  |  |  |  |  |  |  |  |  |  |  |  |  |  |
| 1=No problems | – | – | – | – | – | – | – | 262 | 0.7 (0.7) | 0.0 | 0.0 | 0.6 | 1.0 | 1.9 |
| 2=Slight problems | – | – | – | – | – | – | – | 438 | 1.2 (0.7) | 0.1 | 0.9 | 1.0 | 1.6 | 2.0 |
| 3=Moderate problems | – | – | – | – | – | – | – | 240 | 1.7 (0.6) | 1.0 | 1.3 | 1.9 | 2.0 | 2.3 |
| 4=Severe problems | – | – | – | – | – | – | – | 62 | 2.2 (0.7) | 1.1 | 1.9 | 2.2 | 2.9 | 3.0 |
| 5=Extreme problems | – | – | – | – | – | – | – | 7 | 2.2 (1.0) | 0.1 | 1.7 | 2.6 | 3.0 | 3.0 |

Abbreviations: AP, Abdominal Pain; IBDQ, Inflammatory Bowel Disease Questionnaire; PRO, Patient Reported Outcome; Q, quartile; SD, standard deviation; SF, Stool Frequency.

## Supplementary Table 10. Anchor-based estimates for the PRO (SF and AP) items by supplemental anchors at Week 52.

| Anchors | SF item | | | | | | | AP item | | | | | | |
| --- | --- | --- | --- | --- | --- | --- | --- | --- | --- | --- | --- | --- | --- | --- |
|  | N | Mean (SD) | Percentile | | | | | N | Mean (SD) | Percentile | | | | |
|  |  |  | 10^th^ | 25^th^ (Q1) | 50^th^ (Q2) | 75^th^  (Q3) | 90^th^ |  |  | 10^th^ | 25^th^ (Q1) | 50^th^ (Q2) | 75^th^  (Q3) | 90^th^ |
| IBDQ remission |  |  |  |  |  |  |  |  |  |  |  |  |  |  |
| Yes: ≥170 | 564 | 1.3 (1.7) | 0.0 | 0.1 | 0.7 | 2.0 | 3.6 | 564 | 0.6 (0.6) | 0.0 | 0.0 | 0.4 | 1.0 | 1.3 |
| No: <170 | 271 | 3.1 (2.8) | 0.3 | 1.3 | 2.7 | 4.0 | 6.1 | 271 | 1.2 (0.7) | 0.1 | 0.9 | 1.1 | 2.0 | 2.0 |
| IBDQ Bowel Symptom domain response |  |  |  |  |  |  |  |  |  |  |  |  |  |  |
| ≥8-point increase | 680 | 1.6 (2.0) | 0.0 | 0.1 | 0.9 | 2.3 | 4.0 | – | – | – | – | – | – | – |
| <8-point increase | 153 | 3.2 (2.8) | 0.3 | 1.4 | 2.9 | 4.3 | 6.3 | – | – | – | – | – | – | – |
| IBDQ item 1 (bowel movement frequency) |  |  |  |  |  |  |  | – | – | – | – | – | – | – |
| 7=Normal, no increase in frequency of bowel movements | 461 | 1.2 (1.5) | 0.0 | 0.0 | 0.6 | 1.7 | 3.3 | – | – | – | – | – | – | – |
| 6=Slight increase in frequency of bowel movements | 148 | 2.1 (2.2) | 0.1 | 0.6 | 1.6 | 2.9 | 4.3 | – | – | – | – | – | – | – |
| 5=Some increase in frequency of bowel movements | 77 | 2.4 (1.7) | 0.3 | 1.0 | 2.1 | 3.7 | 4.7 | – | – | – | – | – | – | – |
| 4=Moderate increase in frequency of bowel movements | 71 | 2.6 (1.9) | 0.4 | 1.0 | 2.1 | 3.7 | 5.0 | – | – | – | – | – | – | – |
| 3=Very frequent | 50 | 5.4 (4.1) | 2.0 | 3.1 | 4.5 | 6.3 | 9.9 | – | – | – | – | – | – | – |
| 2=Extremely frequent | 11 | 3.9 (2.4) | 1.7 | 2.0 | 3.4 | 5.3 | 6.9 | – | – | – | – | – | – | – |
| 1=Bowel movements as or more frequent than they have ever been | 17 | 3.5 (2.6) | 0.0 | 1.0 | 3.6 | 6.0 | 7.0 | – | – | – | – | – | – | – |
| IBDQ item 13 (abdominal pain frequency) |  |  |  |  |  |  |  |  |  |  |  |  |  |  |
| 7=None of the time | – | – | – | – | – | – | – | 184 | 0.3 (0.5) | 0.0 | 0.0 | 0.0 | 0.2 | 1.0 |
| 6=Hardly any of the time | – | – | – | – | – | – | – | 250 | 0.6 (0.6) | 0.0 | 0.0 | 0.5 | 1.0 | 1.1 |
| 5=A little of the time | – | – | – | – | – | – | – | 194 | 0.9 (0.6) | 0.1 | 0.4 | 1.0 | 1.1 | 2.0 |
| 4=Some of the time | – | – | – | – | – | – | – | 145 | 1.3 (0.7) | 0.4 | 1.0 | 1.3 | 2.0 | 2.0 |
| 3=A good bit of the time | – | – | – | – | – | – | – | 37 | 1.5 (0.5) | 1.0 | 1.0 | 1.4 | 2.0 | 2.1 |
| 2=Most of the time | – | – | – | – | – | – | – | 16 | 1.6 (0.7) | 1.0 | 1.2 | 1.5 | 2.1 | 2.5 |
| 1=All of the time | – | – | – | – | – | – | – | 9 | 1.6 (0.8) | 0.0 | 1.3 | 1.6 | 2.0 | 3.0 |
| EQ-5D-5L Pain/Discomfort |  |  |  |  |  |  |  |  |  |  |  |  |  |  |
| 1=No problems | – | – | – | – | – | – | – | 378 | 0.5 (0.6) | 0.0 | 0.0 | 0.1 | 1.0 | 1.1 |
| 2=Slight problems | – | – | – | – | – | – | – | 325 | 0.9 (0.6) | 0.0 | 0.4 | 1.0 | 1.1 | 1.7 |
| 3=Moderate problems | – | – | – | – | – | – | – | 120 | 1.5 (0.7) | 0.2 | 1.1 | 1.7 | 2.0 | 2.3 |
| 4=Severe problems | – | – | – | – | – | – | – | 9 | 1.5 (0.7) | 0.0 | 1.0 | 1.9 | 2.0 | 2.3 |
| 5=Extreme problems | – | – | – | – | – | – | – | 3 | 0.8 (0.7) | 0.1 | 0.1 | 0.7 | 1.6 | 1.6 |

Abbreviations: AP, Abdominal Pain; IBDQ, Inflammatory Bowel Disease Questionnaire; PRO, Patient Reported Outcome; Q, quartile; SD, standard deviation; SF, Stool Frequency.


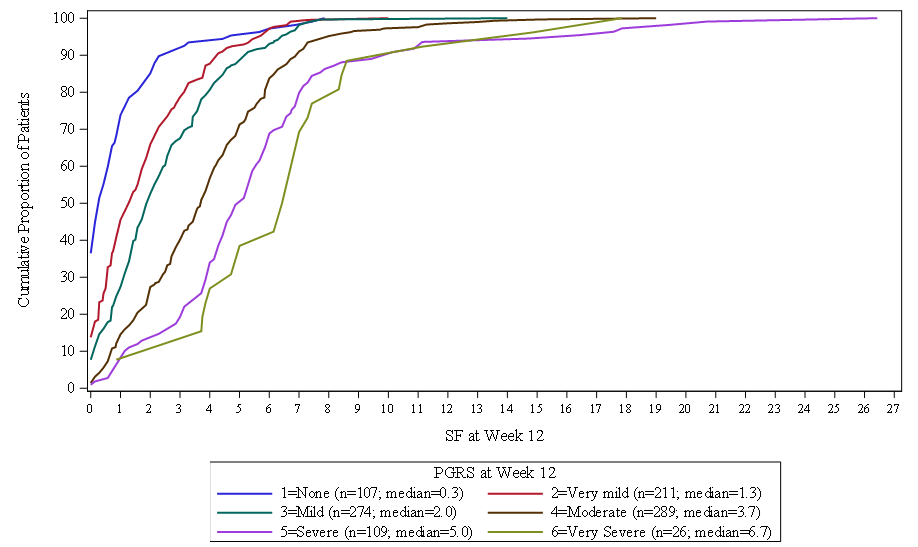

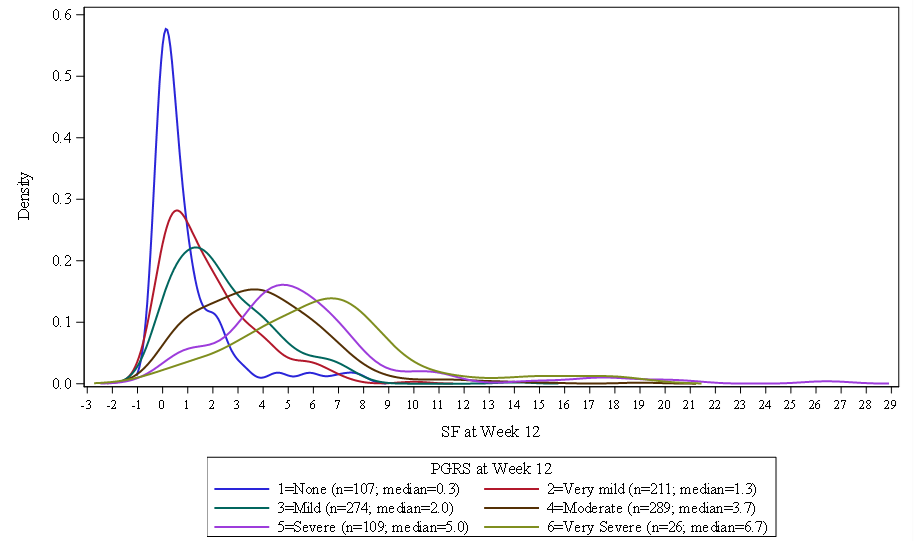


## Supplementary Figure 1. CDF (top) and PDF (bottom) of SF by PGRS at Week 12.

Abbreviations: CDF, Cumulative Distribution Function; PDF, Probability Density Function; PGRS, Patient Global Rating of Severity; SF, Stool Frequency.


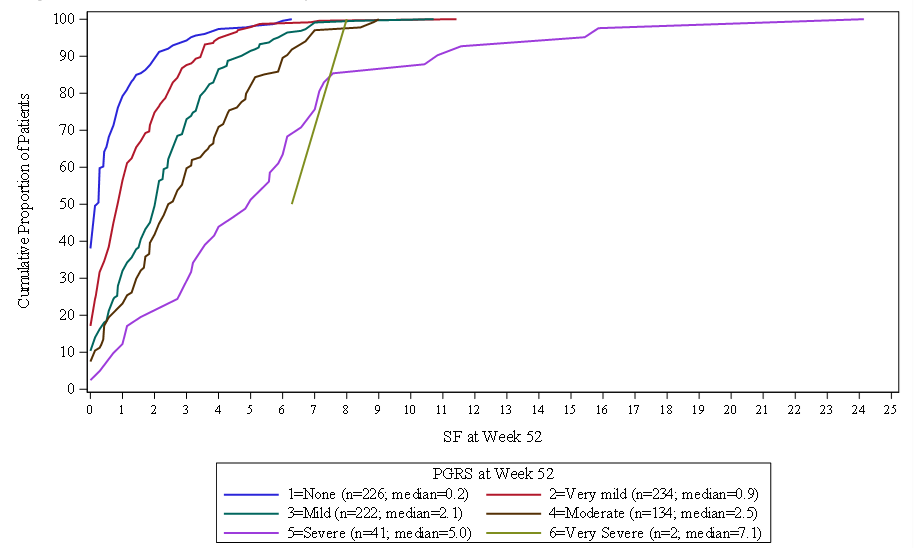

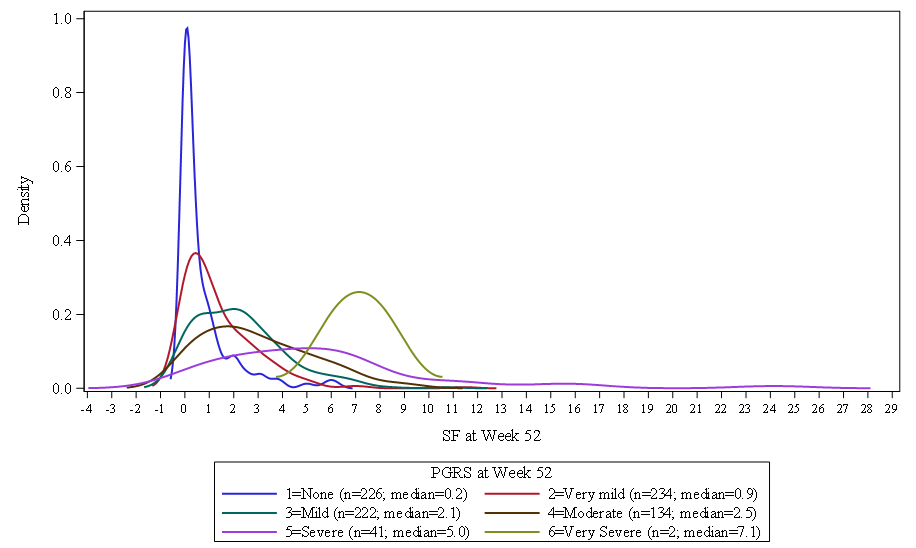


## Supplementary Figure 2. CDF (top) and PDF (bottom) of SF by PGRS at Week 52.

Abbreviations: CDF, Cumulative Distribution Function; PDF, Probability Density Function; PGRS, Patient Global Rating of Severity; SF, Stool Frequency.


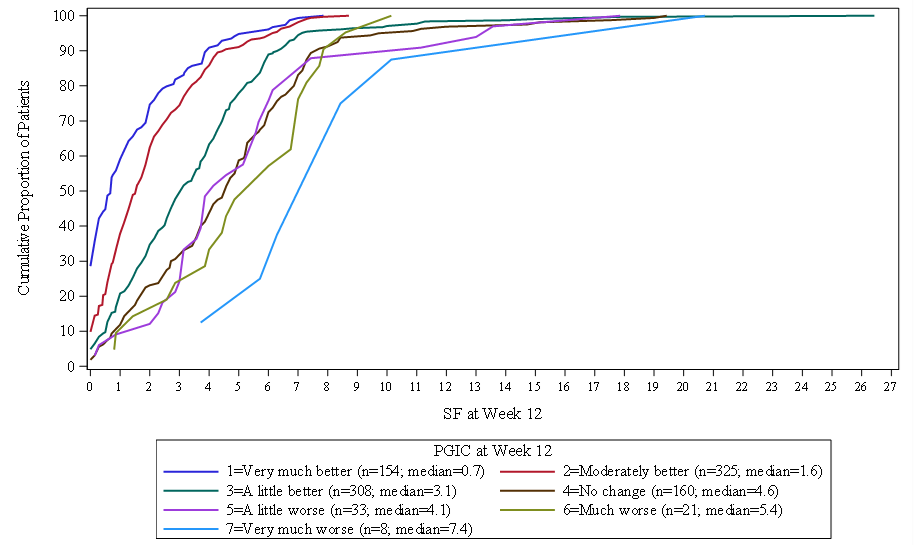


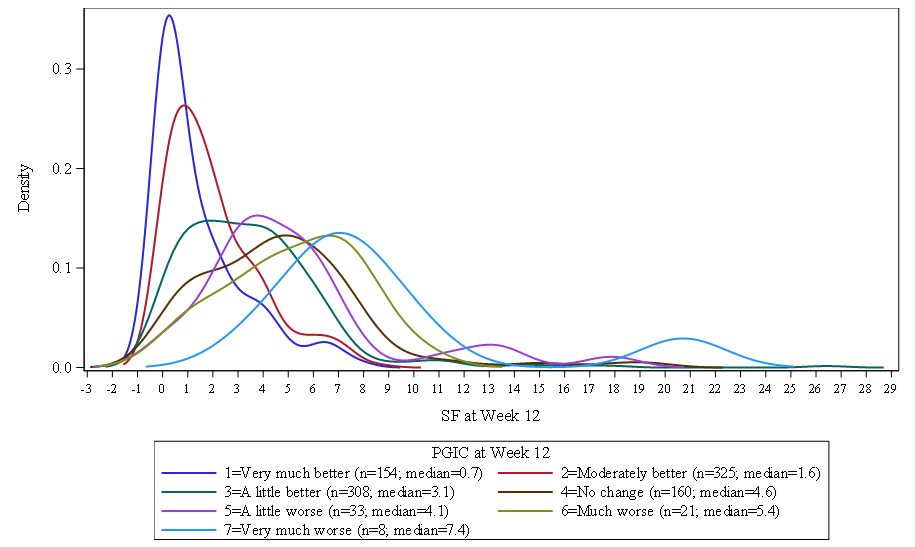


## Supplementary Figure 3. CDF (top) and PDF (bottom) of SF by PGIC at Week 12.

Abbreviations: CDF, Cumulative Distribution Function; PDF, Probability Density Function; PGIC, Patient Global Impression of Change; SF, Stool Frequency.


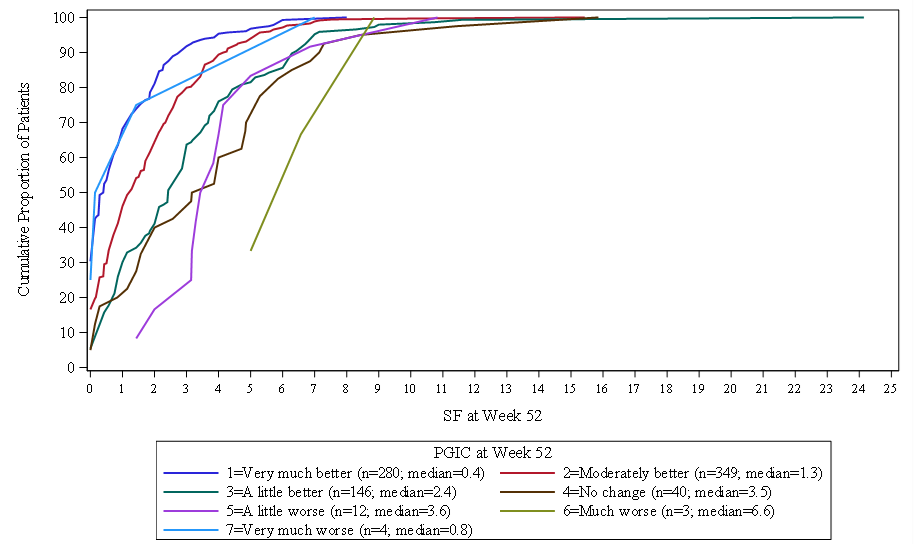

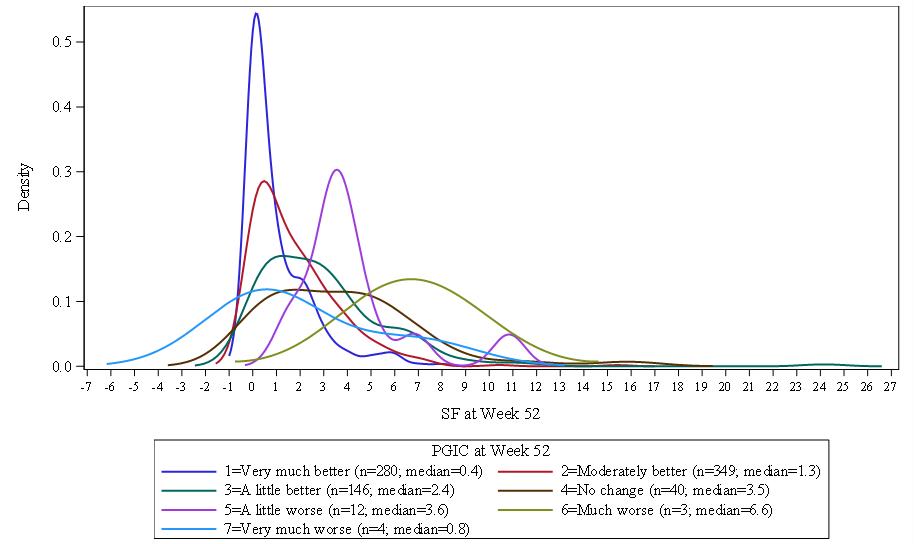


## Supplementary Figure 4. CDF (top) and PDF (bottom) of SF by PGIC at Week 52.

Abbreviations: CDF, Cumulative Distribution Function; PDF, Probability Density Function; PGIC, Patient Global Impression of Change; SF, Stool Frequency.


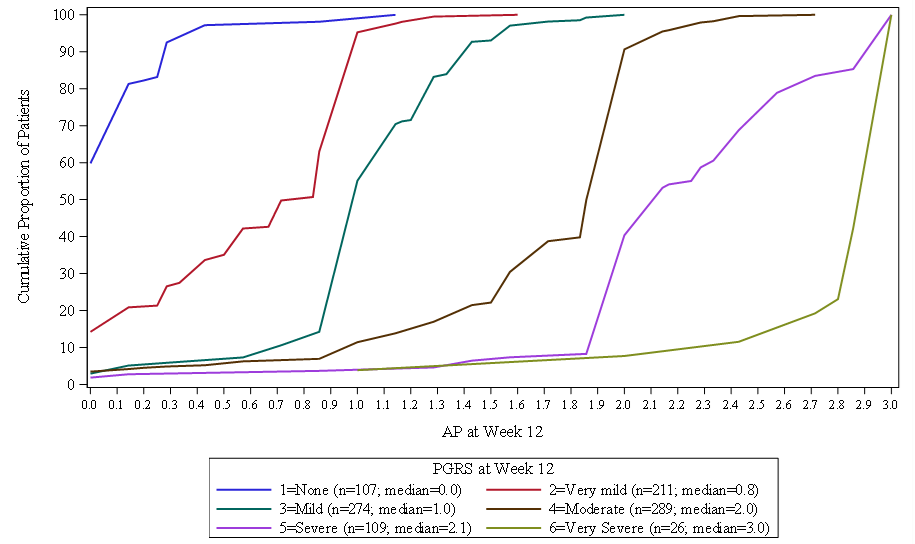

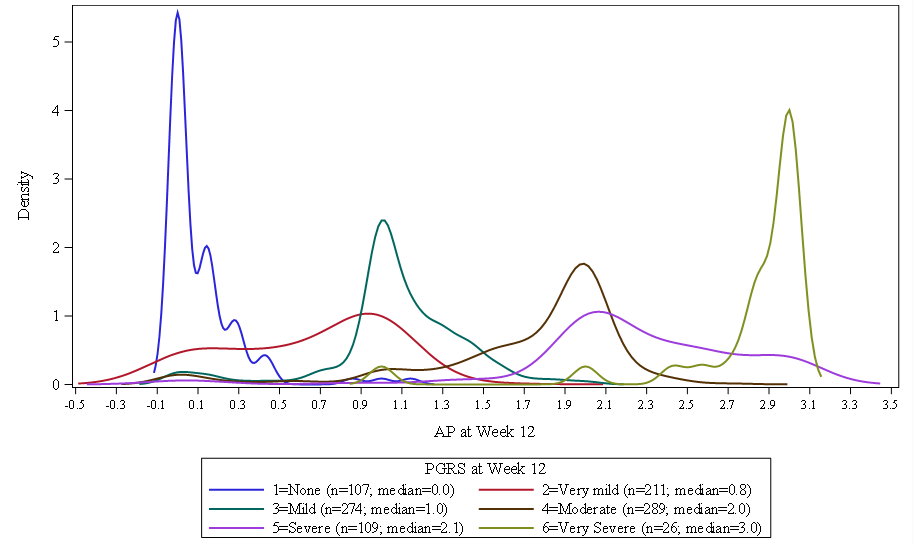


## Supplementary Figure 5. CDF (top) and PDF (bottom) of AP by PGRS at Week 12.

Abbreviations: AP, Abdominal Pain; CDF, Cumulative Distribution Function; PDF, Probability Density Function; PGRS, Patient Global Rating of Severity.


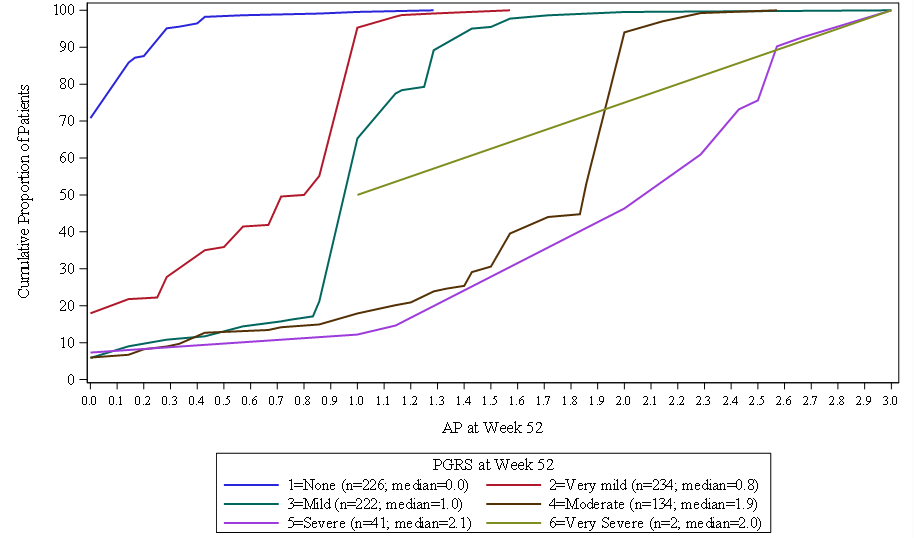


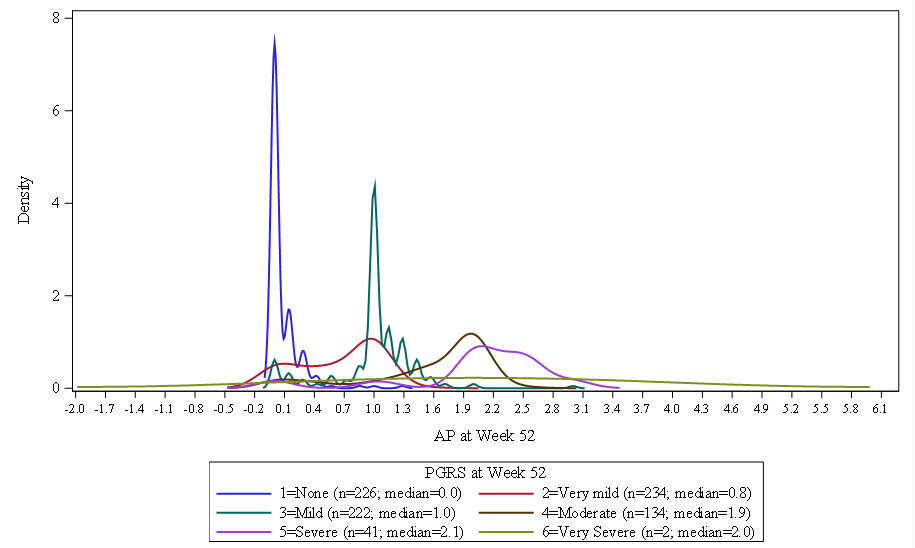


## Supplementary Figure 6. CDF (top) and PDF (bottom) of AP by PGRS at Week 52.

Abbreviations: AP, Abdominal Pain; CDF, Cumulative Distribution Function; PDF, Probability Density Function; PGRS, Patient Global Rating of Severity.


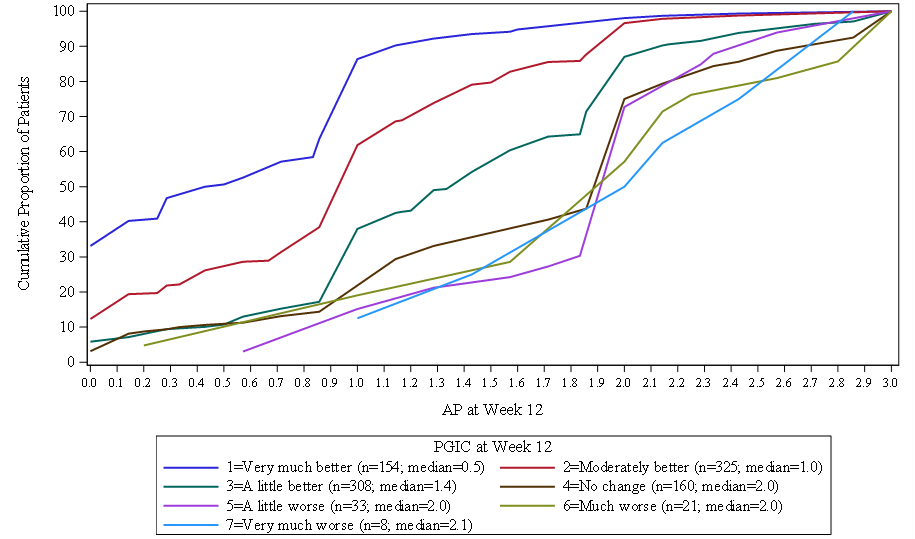


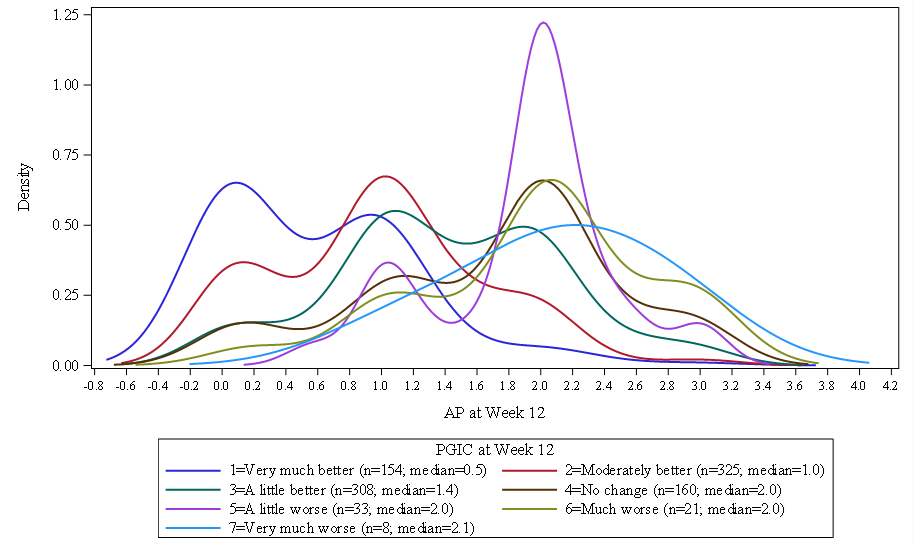


## Supplementary Figure 7. CDF (top) and PDF (bottom) of AP by PGIC at Week 12.

Abbreviations: AP, Abdominal Pain; CDF, Cumulative Distribution Function; PDF, Probability Density Function; PGIC, Patient Global Impression of Change.


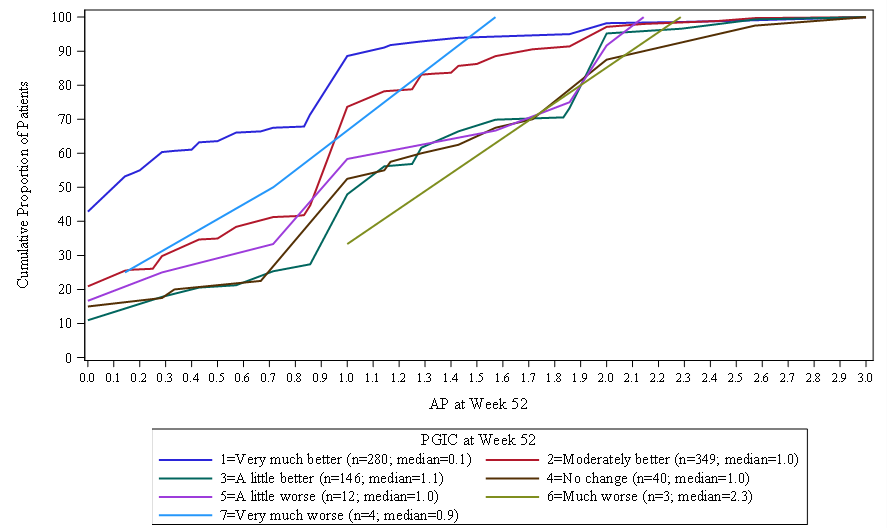


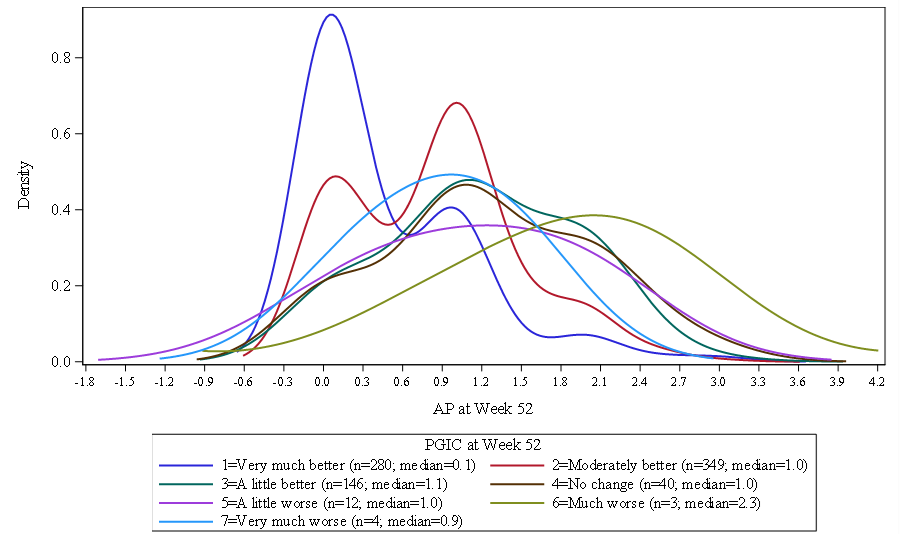


## Supplementary Figure 8. CDF (top) and PDF (bottom) of AP by PGIC at Week 52.

Abbreviations: AP, Abdominal Pain; CDF, Cumulative Distribution Function; PDF, Probability Density Function; PGIC, Patient Global Impression of Change.
